# CD Molecules Nomenclature 2025: Antibody Validation and Expression Profiling of Immune System G Protein‐Coupled Receptors

**DOI:** 10.1002/eji.70099

**Published:** 2025-12-20

**Authors:** Javier Fernández‐Calles, Daniela Kužílková, Fanny Hedin, Violeta Bakardjieva‐Mihaylova, Karolina Škvárová Kramarzová, Menno C. van Zelm, Antonio Cosma, Tomas Kalina, Pablo Engel, the Human Cell Differentiation Molecules (HCDM) organization

**Affiliations:** ^1^ Faculty of Medicine and Health Sciences, Biomedical Sciences University of Barcelona Spain; ^2^ CLIP (Childhood Leukaemia Investigation Prague), Second Faculty of Medicine, Charles University, Department of Paediatric Haematology and Oncology University Hospital Motol Prague Czech Republic; ^3^ Luxembourg Institute of Health Esch‐sur‐Alzette Luxembourg; ^4^ Dept. Immunology, Erasmus MC University Medical Center Rotterdam The Netherlands; ^5^ Dept. Immunology School of Translational Medicine Monash University Melbourne Australia

**Keywords:** monoclonal antibodies, CD molecules, seven‐span cell‐surface molecules, GPCRs, expression profiling, antibody validation

## Abstract

Monoclonal antibodies (mAbs) targeting cell‐surface molecules are pivotal in biomedical research, diagnostic applications, and biotechnology. Over the past four decades, the CD nomenclature system, established by the Human Leukocyte Differentiation Antigens Workshops and endorsed by the International Union of Immunological Societies (IUIS), has provided a standardized naming convention for both mAbs and the cell surface molecules they target. G protein‐coupled receptors (GPCRs) represent the largest family of cell‐surface receptors, playing essential roles in both innate and adaptive immune responses. Despite their significance, GPCRs are underrepresented in terms of well‐validated mAbs available for flow cytometry and therapeutic applications. At the Eleventh HLDA Workshop (HLDA11), new CD nomenclature has been assigned to thirteen GPCR cell‐surface molecules expressed on immune cells: CD198 (CCR8), CD199 (CCR9), CD372 (CCR10), CD373 (CX3CR1), CD374 (XCR1), CD375 (GPR15), CDw376 (GPR26), CD377 (SSTR3), CD378 (C3AR1), CDw379 (FPR2), CD380 (LTB4R), CDw381 (GPR183), and CDw382 (F2RL1). In this article, we introduce the newly established CD nomenclature for mAbs targeting the GPCR family. We detail the quantitative expression profiles of these molecules on various subsets of leukocytes and provide validation data for these mAbs. The implications of these expression profiles are discussed for the potential therapeutic targeting of immune‐mediated diseases and cancer.

## Graphical abstract

HLDA Workshops were established 40 years ago to develop a standardized CD molecule nomenclature for leukocyte cell‐surface molecules by comparing monoclonal antibodies (mAbs) produced by different laboratories and companies. The HLDA11 Workshop focused on validating G‐protein‐coupled receptors (GPCRs) targeting mAbs and assessing cell‐type‐specific expression patterns in leukocytes.

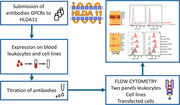

**Abbreviations**
ABC: antibody binding capacity
AML: acute myeloid leukemia
Bdn: double negative B cell
Bnaive: naive B cells
Bnateff: natural effector B cells
BswMem: switched memory B cells
classMono: classical monocytes
CD: cluster of differentiation
CM: central memory
CNS: central nervous system
DAMPs: damage‐associated molecular patterns
EBI2: Epstein–Barr virus‐induced gene 2
EM: effector memory
FMO: fluorescence minus one
GPCRs: G protein‐coupled receptors
HCDM: human cell differentiation molecules
HLDA11: Eleventh Human Differentiation Antigen Workshop
IBD: inflammatory bowel disease
ILC: innate lymphoid cells
interMono: intermediate monocytes
IUIS: International Union of Immunological Societies
mAb: monoclonal antibodies
mDC: myeloid dendritic cells
NK: natural killer
noncMono: nonclassical monocytes
PAMPs: pathogen‐associated molecular patterns
PE: phycoerythrin
pDC: plasmacytoid dendritic cells
SSTR3: somatostatin receptor 3
T‐ALL: acute lymphoblastic leukemia
TemRa: terminal effector memory
Tfh: folicular helper T cells
Treg: regulatory T cells

## 1 Introduction

The cell‐surface proteins expressed by leukocytes are particularly important due to their critical role in facilitating communication between immune cells and their environment, which regulates both the innate and adaptive immune responses. These molecules also enable immune cells to recognize pathogens, maintain immune homeostasis, and prevent autoimmune reactions [1, 2].

The cluster of differentiation (CD) nomenclature is a standardized system that provides a universal language for identifying and classifying leukocyte‐surface molecules [3, 4]. The CD designation refers to a group or cluster of mAbs that recognize a specific cell‐surface molecule expressed on the surface of human leukocytes and other cells relevant to the immune system. Additionally, the CD nomenclature is used to name the molecule itself. This nomenclature has been universally adopted by the scientific community, officially approved by the International Union of Immunological Societies [3, 4].

CD molecules serve as crucial cell‐surface markers, facilitating the identification and isolation of leukocyte populations, subsets, and differentiation stages. MAbs against these cell‐surface molecules have proven essential as biomarkers in diagnostics and research. More recently, these antibodies have been demonstrated to be invaluable tools in immunotherapy for treating a variety of diseases, including cancers, autoimmune disorders, and infectious diseases [5, 6].

The international HLDA/HCDM workshops have standardized and organized the nomenclature of leukocyte surface molecules over the past 40 years. The primary objective of these workshops is to identify, assign, and characterize new CD markers on immune cells, facilitating the communication and collaboration across laboratories worldwide, ensuring that research findings and clinical interventions can be consistently applied and compared across different settings. In clinical diagnostics and treatment, the CD system is indispensable for flow cytometry used to analyze immune cell populations, enabling the widespread use of reliable mAbs [7]. There are more than 400 CD molecules identified to date, but these CD molecules represent only a fraction of the total number of plasma membrane proteins expressed by leukocytes. In fact, it has been estimated that there are over 1000 leukocyte cell‐surface molecules in total [8].

G protein‐coupled receptors (GPCRs) are encoded by the largest gene family in humans, encompassing more than 800 members. These cell‐surface receptors bind to a large variety of endogenous ligands, including hormones, chemokines, and microbial products, which control many physiological processes [9]. In the immune system, GPCRs orchestrate immune surveillance and inflammation by mediating cellular migration and responses to external signals [10, 11]. Thus, GPCRs are considered valuable therapeutic targets in treating a large range of immune‐mediated diseases [12, 13]. GPCRs have a common structure of seven transmembrane α‐helical segments joined with three intracellular and three extracellular loops, an extracellular N‐terminus, and an intracellular C‐terminus [14]. Upon ligand binding, GPCRs activate intracellular G proteins, which initiate signaling cascades. Regardless of their structural similarities, GPCRs have prominent differences in their extracellular and intracellular loops, and these regions are important for ligand binding and interaction with downstream signaling molecules. Moreover, the many GPCRs have unique patterns of expression [15].

Despite being the most abundant family of leukocyte cell‐surface molecules [16, 17], the number of assigned CDs to GPCRs is relatively small compared with those of the immunoglobulin superfamily [8]. Several factors contribute to this shortage, including the low expression levels of GPCRs on blood cells and their unique structure. Consequently, the production of antibodies that recognize the natural, endogenous GPCR proteins is particularly challenging [18].

At the Eleventh HLDA Workshop (HLDA11), new CD nomenclature was assigned to 13 GPCRs expressed on the surface of immune cells: CD198 (CCR8), CD199 (CCR9), CD372 (CCR10), CD373 (CXCR1), CD374 (XCR1), CD375 (GPR15), CDw376 (GPR26), CD377 (SSTR3), CD378 (C3AR1), CDw379 (FPR2), CD380 (LTB4R), CDw381 (GPR183), and CDw382 (F2RL1). In this article, we discuss the expression profiles of these molecules on various subsets of innate and adaptive leukocytes, and provide validation data of these mAbs.

## 2 Results and Discussion

**FIGURE 1:**
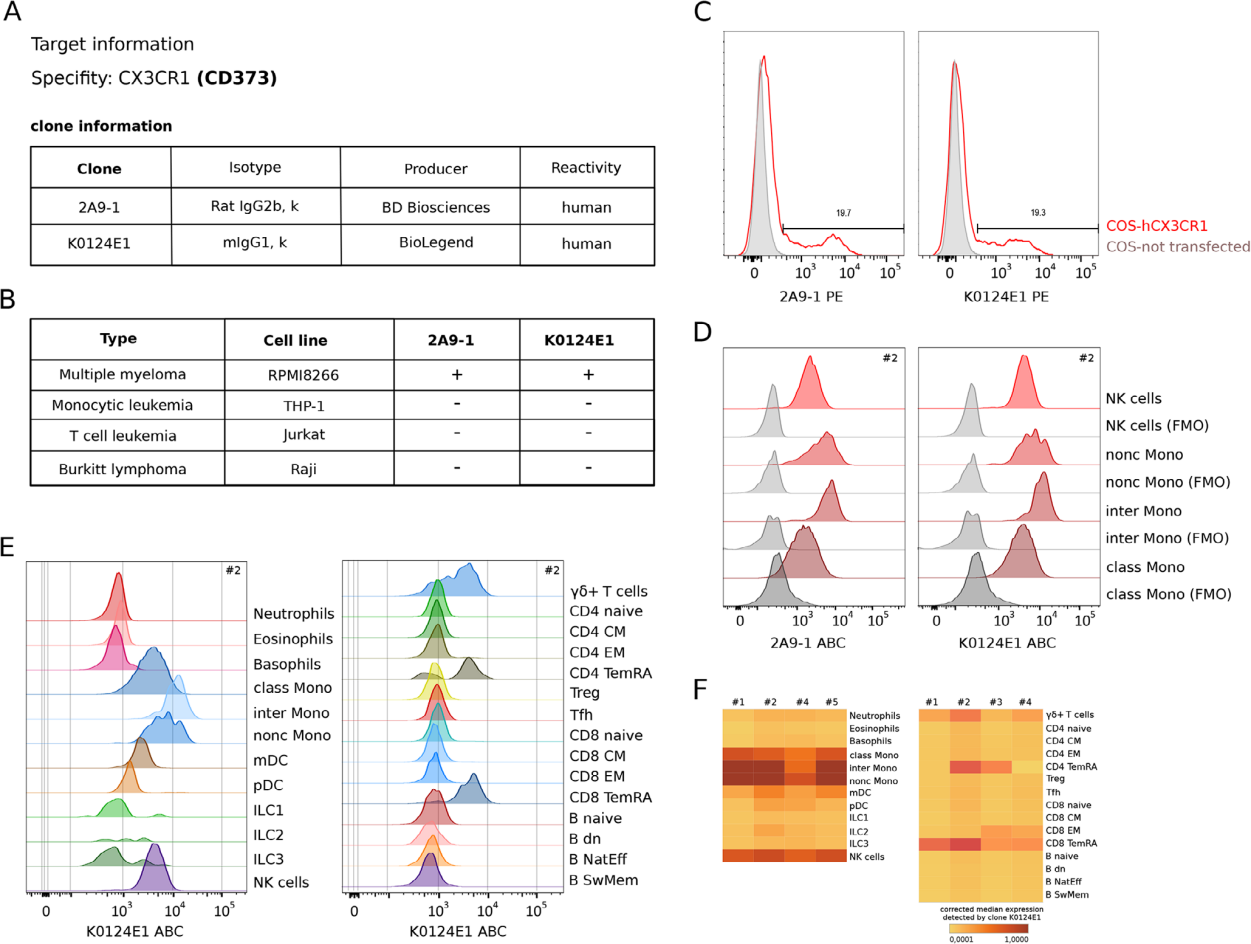
Validation data for CD373 mAbs. (A) List of clones, their isotypes, producers, and reactivities. (B) Reactivity of validated clones with cell lines (second row) representing different cell types (first row). “+” indicates reactivity of particular clone with the respective cells line; “−” indicates no reactivity. (C) Reactivity of individual clones with COS cells transduced with the *CX3CR1* cDNA, red lines indicate transduced cells, grey lines indicate nontransduced cells. The *x*‐axis shows fluorescence intensity of PE. (D) Reactivity of individual clones with selected peripheral blood subpopulations. Red lines represent positive cell subpopulations; gray lines represent FMO controls. The *x*‐axis shows the ABC. (E) Reactivity of K0124E1 clone across 12 innate (left) and 15 adaptive (right) peripheral blood subpopulations. The *x*‐axis shows the ABC. (F) Arcsinh‐transformed, background‐corrected median expression detected by K0124E1 clone across 12 innate (left) and 15 adaptive (right) peripheral blood subpopulations (rows) in four healthy donors (columns). ABC—antibody bound per cell; Bdn—double‐negative B cells; Bnaive—naive B cells; Bnateff—natural effector B cells; BswMem—switched memory B cells; classMono—classical monocytes; CM—central memory; EM—effector memory; FMO—fluorescence minus one; h—human; ILC1‐3—innate lymphoid cells 1‐3; interMono—intermediate monocytes; m—mouse; mAb—monoclonal antibodies; mDC—myeloid dendritic cells; NK—natural killer; noncMono—nonclassical monocytes; pDC—plasmacytoid dendritic cells; PE—phycoerythrine; TemRa—terminal effector memory; Tfh—follicular helper T cells; Treg—regulatory T cells.

The results of all CD markers are presented in Supporting Information Figures. Figure 1 presents an example, summarizing the results for CX3CR1 (CD373).

### 2.1 CD198 (CCR8)

Alternative names: TER1, CKRL1, CMKBR8, CMKBRL2, and CY6; Uniprot: P51685.

HLDA11‐validated mAb clone names (supplier): L263G8 and L263G10 (Biolegend).

#### 2.1.1 Structure and Ligands

This molecule belongs to the beta chemokine receptor family, and its gene is located in the chemokine receptor gene cluster region. It presents the canonical seven‐span structure of chemokine receptors. CCL1, also known as SCYA1 and I‐309, is the best‐known ligand for CCR8. CCR8 has also been shown to function as an alternative coreceptor with CD4 for HIV‐1 infection [19]. In addition. Some viral encoded ligands of CCR8, such as pox‐viral chemokine MC148 and the human herpes virus 8 vMIP‐I, have been shown to be key players for immune evasion [20].

#### 2.1.2 Expression Data

The expression of CCR8 on peripheral blood lymphocytes was quite low (Figure S1F). Our expression data confirmed its expression on peripheral blood T helper cells (Figure S1E). High expression of CCR8 has been described on T cells in several tissues, including the thymus, gut, and skin [21, 22]. We detected very low expression on peripheral blood Treg (Figure S1E), although its presence has been detected in activated Tregs present in tissues, especially in the tumor environment [22]. We also observed a substantial expression on dendritic cells, especially on mDCs (Figure S1D). Accordingly, it has been described that CCR8 regulates the migration of DCs from the skin to the draining lymph nodes [23]. Monocytes and especially neutrophils also expressed amounts of this receptor (Figure S1E).

#### 2.1.3 Function

CCR8 regulates the migration of immune cells, particularly T cells and macrophages, toward sites of inflammation or injury [24]. CCR8 also maintains homeostasis in tissues such as the skin and lungs, contributing to immune surveillance and response to allergens or pathogens [22].

#### 2.1.4 Therapeutic Potential

CCR8 represents a promising target for cancer immunotherapy by disrupting the recruitment of Tregs into tumors, leading to a more favorable tumor microenvironment for immune‐mediated tumor destruction [25]. Because CCR8 is not highly expressed by conventional T cells, it offers a relatively specific treatment approach, potentially minimizing off‐target effects [26]. CD198 antibodies could complement immune checkpoint inhibitors to improve cancer patient outcomes.

### 2.2 CD199 (CCR9)

Alternative names: GPR28; Uniprot: P51686.

HLDA11‐validated mAb clone names (supplier): L053E8 (Biolegend) and CCR9.1.349 (Engel).

#### 2.2.1 Structure and Ligands

CCR9 is a G‐protein‐coupled chemokine receptor primarily involved in the migration and homing of immune cells, especially of immature CD4+CD8+ double‐positive thymocytes and TCRγδ T cells. Its natural ligand is CCL25 (TECK), which is highly expressed by stromal cells of the thymus and gut. Thus, CCR9 regulates thymic development and migration of intraepithelial lymphocytes of the small intestine [27].

#### 2.2.2 Expression Data

We observed very low levels of expression on peripheral blood T and B lymphocytes (Figure S2E). In contrast, most small intestinal lymphocytes, and a subset of thymocytes, were previously reported to express CCR9 [28, 29]. CCR9 is also shown to be highly expressed on CD4+ T‐cell acute lymphoblastic leukemia (T‐ALL) cells [30, 31, 32]. CCR9 was selectively recognized on MOLT4 cells (Figure S2D) by the HLDA11 validated CD199 antibodies.

#### 2.2.3 Function

Being expressed on immature T lymphocytes and intestinal lymphocytes, CCR9 plays a role in T lymphocyte development and the lymphocyte homing to gut‐associated lymphoid tissues in both homeostatic and inflammatory conditions [29, 33].

#### 2.2.4 Therapeutic Potential

The high levels of expression of CCR9 on T‐ALL make this molecule an attractive target for antibody‐based therapies [32, 34]. Additionally, CCR9 is implicated in inflammatory diseases such as Crohn's disease and celiac disease [33]. Thus, it is a potential therapeutic target for both cancer and autoimmune diseases.

### 2.3 CD372 (CCR10)

Alternative names: GPR2 and CC‐CKR‐10; Uniprot: P46092.

HLDA11‐validated mAb clone names (supplier): 1B5 (BD) and 314305 (R&D Systems).

#### 2.3.1 Family and Subfamily

CCR10 is a member of the G‐protein‐coupled receptor 1 family of chemokine receptors. Its natural ligands are CCL27 and CCL28, which are highly expressed in cutaneous and mucosal tissues. It induces chemotaxis of B and T cells by transducing signals that increase intracellular calcium mobilization [35, 36].

#### 2.3.2 Expression Data

As with CCR8 and CCR9, CCR10 was absent from most blood leukocytes (Figure S3E) and was only detected on subsets of CD4+ cells, including memory cells and Tregs (Figure S3D,F). It has been shown that CCR10 is expressed on skin‐homing T lymphocytes in response to inflammation in cutaneous sites and the oral mucosa [35].

In the workshop, CCR10 expression was detected on the myeloma cell line U266 (Figure S3B). We also observed heterogeneous expression levels on plasma cells with clone 1B5, but only very low levels with clone 314305 (Figure S3D).

While both clones (1B5 and 314305) successfully bind to COS cells transduced with *CCR10* cDNA (Figure S3C), 314305 shows lower staining intensity (a 3.8‐fold difference in median signal intensity of plasma B cells, Figure S3D).

CCR10 has previously been detected in malignant plasma cells and proposed to be a prognostic biomarker [37]. CCR10 is also frequently overexpressed in several nonhematological cancers, such as melanoma, breast, and colorectal cancers [38]. Our data also indicate that CCR10 could be expressed on eosinophils (Figure S3E).

#### 2.3.3 Function

CCR10 is vital for guiding immune cells to their target tissues, playing an essential role in both immune defense and the pathology of immune‐mediated diseases [39]. Moreover, CCR10, along with its ligand CCL28, directs plasma cell migration, especially to mucosal surfaces where they produce immunoglobulin A (IgA), a crucial antibody for mucosal immunity [40]. Moreover, IgA‐secreting cells are recruited to mucosal tissues by CCL28 signaling through CCR10 [41].

#### 2.3.4 Therapeutic Potential

Most skin‐infiltrating lymphocytes in patients suffering from psoriasis, atopic, or allergic‐contact dermatitis express CCR10 [35]. Targeting CCR10 could modulate this immune cell migration, offering a strategy to reduce inflammation in these conditions. Blocking CCR10 signaling could also be beneficial in treating chronic inflammatory conditions in mucosal tissues, such as inflammatory bowel disease [38]. In addition to skin diseases, targeting CCR10 has potential in cancer therapy. Cells from several tumors, including melanoma and breast cancer, exploit CCR10‐mediated pathways to metastasize to the skin and mucosal surfaces [42]. Thus, cancer therapies aimed at blocking metastasis have been designed. Given its role in plasma cell migration and retention, CCR10 is also a potential therapeutic target in multiple myeloma [43]. Blocking CCR10 or its ligands has the potential to disrupt the localization and survival of malignant plasma cells, thereby limiting disease progression [44].

### 2.4 CD373 (CX3CR1)

Alternative names: GPR13, CCRL1, CMKBRL1, CMKDR1, and V28; Uniprot: P49238.

HLDA11‐validated mAb clone names (supplier): K0124E1 (Biolegend) and 2A9‐1 (BD).

#### 2.4.1 Family and Subfamily

G‐protein coupled receptor 1 family chemokine receptor. Its natural ligand is CX3CL1, also known as fractalkine. CX3CR1 binds to both the soluble and membrane‐bound forms. CX3CR1 activation by CX3CL1 leads to MAPK and AKT signaling [45].

#### 2.4.2 Expression Data

Our data confirm the previously reported high expression of this chemokine receptor on NK cells and monocytes (Figure 1E) [46]. Although all monocytes expressed CX3CR1, the highest levels were observed on intermediate monocytes (Figure 1D). High levels of this molecule were also found on mDCs, with lower levels on pDCs (Figure 1D). Among lymphocyte subsets, expression of CX3CR1 was selectively very high on Tγ/δ and CD8 TemRA cells (Figure 1E). We also detected expression of CX3CR1 on myeloma cell lines, such as RPMI8226 (Figure 1B), but with very low expression on primary plasma cells (data not shown). Several types of nonhematopoietic cancers, including breast, prostate, pancreatic, renal cell, glioblastoma, and lung, have been reported to express CX3CR1 [47].

#### 2.4.3 Function

The interaction between CX3CL1 and CX3CR1 promotes the adhesion and migration of leukocytes to endothelial cells, facilitating their migration into tissues during immune surveillance and inflammatory responses, recruiting immune cells to sites of injury or infection [48]. In mice, CX3CR1 has also been shown to play a key role in brain microglia by regulating inflammatory response [49].

Inflammatory conditions such as atherosclerosis, inflammatory bowel disease, and multiple sclerosis have been linked to dysregulation of the CX3CR1 pathway [46]. There is growing evidence that the CX3CR1‐CX3CL1 axis also plays a role in cancer metastasis, particularly in cancers that spread to the bones, brain, and lungs [45].

#### 2.4.4 Therapeutic Potential

Given its role in immune cell migration and inflammation, CX3CR1 is a potential therapeutic target for the treatment of chronic inflammatory diseases and neurodegenerative disorders. Particularly in cardiovascular diseases, such as atherosclerosis, CX3CR1 is highly implicated [50]. Blocking the CX3CL1‐CX3CR1 axis in experimental models has been shown to reduce the accumulation of inflammatory cells in arteries, decreasing plaque formation and stabilizing existing plaques [49]. Several inhibitors of the CX3CL1‐CX3CR1 pathway are being explored as treatments for conditions including atherosclerosis, Alzheimer's disease, rheumatoid arthritis, and neuroinflammatory diseases [51]. CX3CR1 expressed on tumor cells aids in their migration to tissues that express high levels of CX3CL1, facilitating metastatic dissemination [52]. Recently, CX3CR1 has also been implicated in the pathogenesis of diffuse parenchymal lung diseases, where its role in immune cell trafficking and persistent inflammation highlights it as a novel potential therapeutic target [53]. However, the targeting of this molecule is complex due to its multiple functions and the wide variety of cells in which it is expressed.

### 2.5 CD374 (XCR1)

Alternative names: GPR5, CCXCR1, lymphotactin receptor; Uniprot: P46094.

HLDA11 validated mAb clone names (supplier): S15046E, S15046A, and S15046K (Biolegend) and 1097A (R&D Systems).

#### 2.5.1 Family and Subfamily

XCR1 is a chemokine receptor that belongs to the G protein‐coupled receptor chemokine family. Its natural ligand is the chemokine XCL1, also known as lymphotactin.

#### 2.5.2 Expression Data

The expression of XCR1 on peripheral blood conventional lymphocytes was very low (Figure S4F). Some expression could be detected on innate lymphocytes and myeloid innate cells (Figure S4E). XCR1 was readily detectable on neutrophils and eosinophils (Figure S4E,F). We also observed detectable levels in myeloma cell lines but not in plasma cells (data not shown). Thus, the role of XCR1 in myeloma cells deserves further exploration. Others have observed the expression and function of XCR1 on cross‐presenting dendritic cells [54, 55]. Although most of the data are obtained from mouse studies, it is clear that in humans XCR1 is selectively expressed by CD141+ DCs and not on lymphocytes or other DC subsets [56, 57]. Recently, XCR1 was shown to be a marker of terminally differentiated cDC1, while XCR1‐cDC1 seems to represent a late immediate precursor of cDC1 [58].

#### 2.5.3 Function

Chemokine–receptor interaction guides XCR1‐expressing DCs to areas where they can recognize, process, and present pathogens to T cells, thereby promoting effective antigen‐specific T cell responses in viral immunity [59]. Beyond antigen presentation, XCR1 signaling also affects the activation and maturation of DCs, playing a key role in regulating both innate and adaptive immunity [60].

#### 2.5.4 Therapeutic Potential

The XCR1+ cross‐presenting DC subset is being investigated as a next‐generation DC therapy due to its specialized ability to prime effector CD8+ T cells and mediate anti‐tumor responses. New protocols aimed at generating large quantities of XCR1+ DCs, rather than conventional mDCs, may offer significant therapeutic advantages [54].

### 2.6 CD375 (GPR15)

Alternative names: GPR15 and Brother of Bonzo (BoB); Uniprot: P49685.

HLDA11‐validated mAb clone names (supplier): SA302A10 (Biolegend) and 367902 (R&D).

#### 2.6.1 Family and Subfamily

GPR15 is a G protein‐coupled receptor that was initially reported as a co‐receptor for human immunodeficiency virus (HIV) and simian immunodeficiency virus (SIV), with structural similarity to other members of the chemoattractant receptor family. Its natural ligand is C10orf99, a chemokine‐like polypeptide, which is strongly expressed by gastrointestinal tissues. GPR15‐C10orf99 is a novel signaling axis that controls intestinal homeostasis and inflammation through the migration of immune cells [61].

#### 2.6.2 Expression Data

In agreement with published data [62], we observed high expression on several T and B lymphocyte subsets (Figure S5E). The highest levels were detected on CD8 Tnaïve, CD8 TemRA, memory B cells, and plasma cells (Figure S5F). The role of GPR15 on these cells remains to be explored.

GPR15 has been shown to be highly expressed on Th17 cells in the peripheral blood of patients with ulcerative colitis or multiple sclerosis [63, 64]. Interestingly, we also observed expression of GPR15 on dendritic cells, with higher expression on pDCs as compared with mDCs (Figure S5E). GPR15 has also been reported to be expressed by endothelial cells [62].

#### 2.6.3 Function

The GPR15‐C10orf99 axis has been reported to control intestinal homeostasis and inflammation through the migration of immune cells [61]. In humans, GPR15 controls, together with α4β7‐integrin, the homing of effector T cells to the inflamed gut of ulcerative colitis [65]. In addition to the gut, GPR15 is also expressed in mucosal surfaces, such as the respiratory tract and skin, associated with the homing of T cells [66].

#### 2.6.4 Therapeutic Potential

One of the primary therapeutic areas of interest for GPR15 is inflammatory bowel disease (IBD), both Crohn's disease and ulcerative colitis, and rheumatoid arthritis. Dysregulation of GPR15 expression has been associated with increased intestinal inflammation, making it a potential target for therapies aimed at restoring immune balance in IBD [63, 67].

Additionally, GPR15 has been explored in the context of cancer. The receptor is overexpressed in certain types of malignancies, such as colorectal cancer, and has been linked to tumor progression. Therapies that inhibit GPR15 signaling may reduce tumor growth and metastasis, offering potential in cancer immunotherapy [68].

### 2.7 CDw376 (GPR26)

Alternative names: none; Uniprot: Q8NDV2.

HLDA11‐validated mAb clone name (supplier): 891318 (R&D Systems).

#### 2.7.1 Family and Subfamily

GPR26 is primarily known as an orphan G protein‐coupled receptor since its endogenous ligand is not well‐characterized [69, 70].

#### 2.7.2 Expression Data

Our data indicate that innate cells, such as neutrophils and monocytes, express this receptor (Figure S6E). GPR26 expression has been reported on activated THP‐1 cells and monocytes from diabetic patients [69]. Expression on both blood T and B lymphocytes could also be observed (Figure S6D). However, we were not able to find any cell line expressing this receptor (Figure S6B).

#### 2.7.3 Function

One report indicates that GPR26 could inhibit proinflammatory monocyte activation, ROS production, and apoptosis [69]. GPR26 has been studied mainly in the context of the central nervous system (CNS) and its potential role in neurological functions, including its involvement in mood regulation, anxiety, and possibly neurodegenerative diseases such as Alzheimer's. It is also expressed in the brain regions related to these functions [71]. GPR26's role in the immune system is not well‐documented.

#### 2.7.4 Therapeutic Potential

Unknown in the context of immune‐mediated diseases.

### 2.8 CD377 (SSTR3)

Alternative names: Somatostatin receptor type 3, SS3R, SST3; Uniprot: P32745.

HLDA11‐validated mAb clone names (supplier): 891318 (R&D Systems) and 7H8E5 (Thermo).

While both clones react similarly with SSTR3‐transfected cells, we observed that clone 7H8E5 exhibits lower staining intensities than 891318 on peripheral blood cells (Figure S7C).

#### 2.8.1 Family and Subfamily

Somatostatin receptor 3 (SSTR3) belongs to the family of GPCRs known as somatostatin receptors. While somatostatin receptors are primarily associated with neuroendocrine functions, they are also expressed on various immune cell types.

#### 2.8.2 Expression Data

We detected expression of SSTR3 on various T cell subsets, especially within the CD8 lineage (Figure S7D,F). This is consistent with observations by others [72], though its functional role is unknown. We detected low levels of expression on innate cells such as neutrophils (Figure S7E). Here, we also report the presence in the myeloma cell line RPMI8226 (Figure S7B), which indicates that it may be expressed on myeloma cells.

While both clones (576017 and 7H8E5) successfully bind to COS cells transduced with *SSTR3* cDNA (Figure S7C), we observed lower staining intensity in the 7H8E5 clone (average 2.8‐fold difference in median signal intensity (range 2.3–3.2) of CD8+ T cell subsets; Figure S7D).

#### 2.8.3 Function

The activation of somatostatin receptors typically results in the inhibition of immune cell proliferation, reduced cytokine production, and suppression of inflammatory responses [17]. The specific function of SSTR3 remains unclear, although it has been speculated that it could help to reduce excessive inflammation and autoimmunity [73].

#### 2.8.4 Therapeutic Potential

The immunosuppressive effects of SSTR3 suggest that it might be a potential target for therapies aimed at reducing inflammation or treating autoimmune or autoinflammatory diseases.

### 2.9 CD378 (C3AR1)

Alternative names: AZ3B, C3R1, C3AR and HNFAG09; Uniprot: Q16581.

HLDA11‐validated mAb clone names (supplier): hC3aRZ8 (BD) and 534625 (Thermo).

#### 2.9.1 Family and Subfamily

C3AR1 is a G protein‐coupled receptor for the anaphylatoxin C3a, a cleavage fragment released by complement activation. This receptor stimulates chemotaxis, granule enzyme release, and superoxide anion production. C3aR1 couples preferentially to Gi/o/z proteins and can recruit β‐arrestins to cause internalization [74].

#### 2.9.2 Expression Data

Our expression data confirm the expression of this complement receptor on myeloid cells, including neutrophils, basophils, eosinophils, and monocytes (Figure S8E,F) [75]. The highest levels were found on eosinophils (Figure S8D). Intermediate and nonclassical monocytes express higher levels of C3AR1 than classical monocytes (Figure S8E). The levels of expression on DCs were lower on monocytes (Figure S8E,F). We did not detect expression on lymphocytes (Figure S8E).

#### 2.9.3 Function

As a receptor for the complement anaphylatoxin C3a, C3AR1 mediates proinflammatory effects. This receptor also induces and stimulates chemotaxis, granule enzyme release, and superoxide anion production [76]. However, in certain pathological conditions, C3aR1 can also contribute to the resolution phase of inflammation by promoting the clearance of apoptotic cells, playing an anti‐inflammatory role. Thus, this complement receptor may exert anti‐inflammatory or proinflammatory effects depending on different cell types and diseases [77].

#### 2.9.4 Therapeutic Potential

C3AR1 has been proposed as a therapeutic target in various conditions, such as acute myeloid leukemia (AML) and immune‐related diseases [77, 78, 79].

### 2.10 CDw379 (FPR2)

Alternative names: N‐formyl peptide receptor 2, LXA4R, ALXR, Lipoxin A4 receptor; Uniprot: P25090.

HLDA11‐validated mAb clone name (supplier): 304405 (R&D Systems).

#### 2.10.1 Family and Subfamily

FPR2 is a low‐affinity receptor for N‐formyl‐methionyl peptides. Together with FPR1 and FPR3, it forms a family of receptors that is involved in antibacterial defense and inflammation. It activates a phosphatidylinositol‐calcium second messenger system via a G‐protein [80, 81].

#### 2.10.2 Expression Data

This receptor has been reported to be expressed on myeloid cells, especially neutrophils [82]. We detected the highest amounts of FPR2 on monocytes and mDCs (Figure S9E). In addition, we observed expression on several T‐ and B‐cell subsets, especially CD8+ T cells (Figure S9D). Its role in these cells remains to be explored.

#### 2.10.3 Function

FPRs are involved in host defense against pathogens via recognition of conserved pathogen‐associated molecular patterns (PAMPs), in particular, N‐formylated peptides. In addition, FPRs sense damage‐associated molecular patterns (DAMPs) to respond to host‐mediated inflammation during noninfectious inflammation [78, 83]. The type of ligand present in the environment determines whether FPR2 triggers inflammation or promotes its resolution. Whereas bacterial formyl peptides (e.g., fMLP) trigger strong proinflammatory responses, other ligands, such as lipoxin A4 and annexin A1, induce the resolution of inflammation and tissue repair [83, 84].

#### 2.10.4 Therapeutic Potential

FPR2 is a promising target for treating inflammatory and autoimmune diseases by harnessing its dual role in inflammation and resolution. However, no antibody‐mediated therapies have been developed so far [85, 86].

### 2.11 CD380 (LTB4R)

Alternative names: Leukotriene 4 receptor, BLTR, BLT1, P2Y7, CMKRL1, GPR16, P2RY7; Uniprot: Q15722.

HLDA11‐validated mAb (clone names): 203_1411 (BD) and 202/7B1 (Serotec).

#### 2.11.1 Family and Subfamily

LTB4R is a chemotactic G protein‐coupled receptor [87]. LTB4R is a receptor for leukotriene B4.

#### 2.11.2 Expression Data

Our results confirm previous studies showing high levels of expression on neutrophils, eosinophils, and monocytes (Figure S10E,F) [88]. We also observe significant differences in expression levels of this receptor on monocyte subsets, with classical monocytes expressing higher levels than nonclassical and intermediate monocytes (Figure S10D). We found very low levels of LTB4R on resting T and B cells (Figure S10E), but we cannot exclude that its expression increases with activation. In addition, LTB4R has been shown to be highly expressed on keratinocytes [89].

#### 2.11.3 Function

LTB4 is a potent chemoattractant involved in inflammation and immune responses. Neutrophils are the primary responders to LTB4 signaling. It facilitates chemotaxis, activation, and recruitment to sites of inflammation. In macrophages, LTB4R plays a role in inflammatory signaling and tissue remodeling [90]. In mice, LTB4R has been shown to play a role in T effector cell migration to inflammatory sites [91].

#### 2.11.4 Therapeutic Potential

Inhibition of LTB4R has been tested for the amelioration of bronchial asthma and allergic rhinitis symptoms, but there are no clinically approved monoclonal antibodies [92].

### 2.12 CDw381 (GPR183)

Alternative names: EBV‐induced G‐protein coupled receptor 2 (EBI2); Uniprot: P32249.

HLDA11‐validated mAb clone name (supplier): SA313E4 (Biolegend).

#### 2.12.1 Family and Subfamily

GPR183, also known as Epstein–Barr virus‐induced gene 2 (EBI2), is a G protein‐coupled receptor that is closely related to the thrombin receptor and forms homodimers and heterodimers when it associates with CXCR5 [93, 94]. Its natural ligands are oxysterols, which are produced by enzymes involved in cholesterol metabolism [95].

#### 2.12.2 Expression Data

We confirm previous reports showing expression of GPR183 on a variety of T and B cell subsets (Figure S11E) [95]. Our results showed lower expression levels on naive B cells as compared with memory B cells (Figure S11F). Moreover, ILC2 expressed this receptor (Figure S11E). CD4 T cells also express substantial amounts of this protein (Figure S11E). Among the myeloid cells, basophils and DCs expressed very high levels of GPR183 (Figure S11E).

#### 2.12.3 Function

GPR183 functions as a chemotactic receptor for B cells and T cells. GPR183 has been shown to regulate B‐cell migration and positioning within secondary lymphoid organs, coordinating germinal center formation and differentiation into plasma cells [96, 97]. In myeloid cells, GPR183 controls the migration and tissue localization of DCs, enhancing T cell priming in inflammatory processes [98]. GPR183 is an Epstein–Barr virus‐induced gene, and its expression has been related to human neoplastic diseases, such as AML, chronic lymphocytic leukemia, and diffuse large B‐cell lymphoma [99].

#### 2.12.4 Therapeutic Potential

The functional characteristics of this receptor make it an interesting therapeutic target for both autoimmunity. Small molecules that antagonize GPR183 have been tested for the treatment of rheumatoid arthritis, but no antibody‐based therapies are currently evaluated in clinical trials [100].

### 2.13 CDw382 (F2RL1)

Alternative names: F2R‐like trypsin receptor 1, PAR2, GPR11, proteinase‐activated receptor‐2, and thrombin receptor‐like 1; Uniprot: P55085.

HLDA11‐validated mAb clone name (supplier): 344222 (R&D Systems).

#### 2.13.1 Family and Subfamily

F2RL1 is a receptor for trypsin and trypsin‐like enzymes. It senses and responds to active proteases in the cellular microenvironment. Activated PAR2 engages multiple G protein‐dependent signaling pathways, including phospholipase C (PLC), intracellular calcium, mitogen‐activated protein kinase (MAPK), and NF‐kappaB.

#### 2.13.2 Expression Data

F2RL1 has been shown to be expressed on keratinocytes, neurons, and some immune cells, including neutrophils and lymphocytes [101]. Our results indicate high expression levels on several T cell subsets (Figure S12E,F). Especially high is the expression on most of the CD8+ population (Figure S12D). Additionally, we also detected the presence of this receptor on B cells, with the highest expression on memory B cells (Figure S12E). Regarding the cells of the innate immunity, we observed the highest expression levels on mDC (Figure S12E).

#### 2.13.3 Function

Despite its high expression on several lymphocyte subsets, relatively little is known of the function of F2RL1 on these cells in humans. It has been shown that mouse F2RL1 can modulate T cell responses, influencing cytokine production and T cell proliferation [102]. On B cells, it may play a role in controlling antibody production [103].

F2RL1 is known to play a role in inflammation of the skin, delaying barrier repair and affecting keratinocyte differentiation. It also actively participates in itch and pain sensations in this tissue [104, 105]. Moreover, other studies have involved this receptor in other pathogenic inflammatory processes such as allergy, asthma, lung injury, inflammatory bowel diseases, and cancer [106].

#### 2.13.4 Therapeutic Potential

So far, no antibody‐mediated therapies have been tested, although this molecule represents an interesting therapeutic target because of its expression pattern and multiple associations with inflammatory processes.

## 3 Concluding Remarks

The HLDA11 studies summarized in this paper have advanced the validation of antibodies against GPCRs. New CD nomenclature has been assigned to thirteen GPCR surface molecules expressed on immune cells (Table 1), and revealed unexpected expression patterns of these molecules on leukocyte subsets. These discoveries will guide future research into the immune functions of GPCRs and their potential as diagnostic and therapeutic targets.

**TABLE 1:** New CD numbers for G‐protein coupled receptor family molecules defined in the HLDA11 workshop.

CD	Gene name	Other names	Gene subfamily	Gene number (HGNC)
CD198	CCR8	TER1, CKRL1, CMKBR8, CMKBRL2, CY6	CC chemokine receptor	1609
CD199	CCR9	GPR‐9‐6, GPR28, chemokine (C‐C Motif) receptor 9	CC chemokine receptor	1610
CD372	CCR10	GPR2, CC‐CKR‐10	CC chemokine receptor	4474
CD373	CX3CR1	V28, CMKBRL1, CMKDR1, CCRL1, GPR13	CX3C chemokine receptor	2558
CD374	XCR1	CCXCR1, GPR5, lymphotactin receptor	XC chemokine receptor	1625
CD375	GPR15	Brother of Bonzo, BoB	Orphan‐A receptor	4469
CDw376	GPR26	—	Orphan‐A receptor	4481
CD377	SSTR3	Somatostatin receptor type 3, SS3R, SST3, SSR28	Somatostatin receptor	11332
CD378	C3AR1	AZ3B, C3R1, C3AR, HNFAG09	Anaphylatoxin receptor	1319
CDw379	FPR2	N‐formyl peptide receptor 2, LXA4R, ALXR, Lipoxin A4 receptor, HM63	Chemoattractant receptor	3827
CD380	LTB4R	Leukotriene 4 receptor, BLTR, BLT1, P2Y7, CMKRL1, GPR16, P2RY7	Chemoattractant receptor	6713
CDw381	GPR183	EBV‐induced G‐protein coupled receptor 2, EBI2, HEBI2	Orphan‐A receptor	3128
CDw382	F2RL1	PAR2, GPR11, f2r like trypsin receptor 1, proteinase‐activated receptor‐2, thrombin receptor‐like 1	Proteinase‐activated receptor	3538

A key outcome of the HLDA11 workshop is the assignment of CD names to all chemokine receptors expressed on leukocytes, providing a standardized framework for further studies. However, some molecules received only provisional CDw designations due to the availability of a single validated antibody, underscoring the need for additional antibody development and characterization to ensure robust validation (Table S3).

The reduced numbering of some CDs reflects the fact that these had previously been designated as provisional CDw in earlier workshops due to the fact that there was only one approved clone. These were only validated and assigned definitive CD status during HLDA11.

Given the critical role of GPCRs in immune regulation and disease, there is an urgent need to expand and validate monoclonal antibodies against these receptors. Increasing the availability of such antibodies will advance our understanding of GPCR functions and enhance their use as biomarkers and therapeutic targets. Future efforts should prioritize the production and refinement of antibodies, unlocking the full potential of GPCRs in immunology and clinical medicine.

## 4 Materials and Methods

### 4.1 Human Blood Samples

The use of blood samples from healthy adults was approved by the Human Ethics Committee of the Motol University Hospital and was contingent on informed consent in accordance with the Declaration of Helsinki. Blood buffy coats were obtained from the Institute of Haematology and Blood Transfusion (Prague, Czech Republic)

### 4.2 Flow Cytometry Experimental Procedure

For the identification of leukocyte subsets, two antibody panels with 10 and 11 fluorescent parameters, respectively, were designed (Table S1). The innate panel (11 parameters) enables the identification of 12 cell subsets, including eosinophils, neutrophils, and basophils, as well as classical, intermediate, and nonclassical monocytes. Additionally, it allows the detection of NK cells, innate lymphoid cells (ILC) 1–3, and plasmacytoid and myeloid dendritic cells (pDC and mDC). The Adaptive panel (10 parameters) enables the identification of 15 cell subsets. Among B cells, it distinguishes four subsets: naïve, natural effector, switched memory, and IgD/CD27 double‐negative (dn) B cells. Within the T‐cell compartment, it identifies naïve, central memory (CM), effector memory (EM), and terminal effector memory (TemRA) CD4 and CD8 T cells, as well as regulatory T cells (Treg), follicular helper T cells (Tfh), and TCRγδ+ T cells. The cytometry method is extensively described in the study by Kužílková et al. [107].

### 4.3 Flow Cytometry Equipment

Data acquisition was performed on the four‐laser conventional LSR II (405, 488, 561, and 647 nm; BD Biosciences, San Jose, CA, USA) and the 5‐laser spectral Aurora (355, 405, 488, and 640 nm; Cytek) cytometer instruments. Both instruments were equipped with a plate loader.

### 4.4 Flow Cytometer Instrument Setup

Cytometer setup and tracking beads (BD Biosciences) and 8‐peak Rainbow bead calibration particles (Spherotech, Lake Forest, IL, USA) were used for PMT voltages and light scatter setup for the BD instrument to achieve interlaboratory standardization as developed by the EuroFlow consortium [108]. The EuroFlow standard operating procedure (SOP) for Instrument Setup and Compensation can be downloaded from www.euroflow.org. SpectroFlo QC beads (Cytek) were used for routine performance tracking of the Cytek instrument. The PE‐conjugated target mAbs were excited by the 561 nm laser (BD instrument) or 488 nm laser (Cytek instrument); for each staining (well), a minimum of 0.5 million events were acquired. All target PE‐conjugated antibodies were titrated as described previously [107]. All data were processed and visualized using Tableau Prep and Tableau Desktop as previously described [109].

### 4.5 Single Cell Isolation and Staining of Blood Leukocytes

The blood leukocyte isolation protocol was optimized to minimize platelet adhesion (satellitism) [110] and performed as described previously [107].

### 4.6 Conversion of PE Fluorescence Intensity to Antibody Binding Capacity

To convert PE fluorescence to the amount of PE molecules bound to a target (antibody binding capacity [ABC] parameter), we used the PE Fluorescence quantitation kit (BD Biosciences) as described previously [107].

### 4.7 Analysis, Gating, and Export of Values

The leukocyte and lymphocyte subsets to be analyzed were predefined and gated using FlowJo (v10, BD Biosciences). The gating strategy was previously shown [107]. For each defined subset, the PE+ gate was set based on the control sample with an empty PE channel (fluorescence minus one; FMO) prepared in parallel to each experiment. An example of background in FMO is presented in Figure S13. From each defined subset, ten statistical parameters of the ABC parameter were extracted: 50th percentile, mean, mode, CV, 10th percentile, 25th percentile, 75th percentile, 90th percentile, count, and percentage of PE+ population. Finally, for each donor and cell subset, the 50th percentile of the ABC intensity from the corresponding FMO control was subtracted. Negative values were replaced with 0.0001. The resulting parameter was named median_corrABC. The minimum cell count for statistical evaluation was set to 25, and subsets with lower cell counts were omitted from further analysis.

Heatmaps were created using Tableau Prep Builder and Tableau Desktop (Salesforce, San Francisco, California, version 2023.2). Values displayed in the heatmaps were calculated as the arcsinh‐transformed median_corrABC with a cofactor of 5000.

The data are presented in concordance with the guidelines for the use of flow cytometry and cell sorting in immunological studies [111].

### 4.8 Cell Lines

Various human cell lines were selected as representatives of the different cell types targeted by the 11th HLDA workshop: HDLM‐2 (Hodgkin lymphoma), THP‐1 (Monocytic leukemia), Jurkat (T cell leukemia), Raji (Burkitt lymphoma), MOLT4 (T cell leukemia), U266 (Multiple myeloma), RPMI8226 (Multiple myeloma), JVM‐2 (Chronic B cell leukemia), U937 (Histiocytic lymphoma) (American Tissue Culture Collection [ATCC], Rockville, MD, USA). Additionally, COS monkey kidney (ATTC) and 300.19 (pre‐B) generated in F. Alt's laboratory (Harvard Medical School, Boston) were used for transfection.

### 4.9 Transfections

COS cell line was transfected with each cDNA in expression vectors using the Nucleofector kit R in an Amaxa Inc I/II/2b (Lonza) (see Table S2). After 24 h, the cells were tested with mAbs using flow cytometry.

## Conflicts of Interest

The authors declare no conflicts of interest.

## Funding

D. K. was supported by the Ministry of Health of the Czech Republic, grant no. NU23J‐03‐00026. The CLIP laboratory was funded by the European Union—Next Generation EU—program no. LX22NPO5102 and by the Charles University Research Centre program no. UNCE/24/MED/003. The National Cytometry Platform is supported by funding from Luxembourg's Ministry of Higher Education and Research (MESR). This work was supported by the Agencia Estatal de Investigación, Spanish Ministry of Science, Innovation and Universities (PID2023‐146941OB‐I00 to Pablo Engel). MCvZ was supported by an NHMRC Ideas grant (2000773) and an Australian MRFF grant (2016108).

## Supporting information




**Supporting File 1**: eji70099‐sup‐0001‐SuppMat.pdf.

## Data Availability

The data supporting the findings of this study are openly available in Tableau Public at https://public.tableau.com/views/dfnysxfgmc35163154154wb/Menu?:language=en‐US&:sid=&:redirect=auth&:display_count=n&:origin=viz_share_link and on hcdm.org (HCDM HCDM/HLDA11 workshop/data repository).
