# Supplementary material for: CD Molecules Nomenclature 2025: Antibody Validation and Expression Profiling of Immune System G Protein‐Coupled Receptors
Source: Eur J Immunol. 2025 Dec 20;55(12):e70099. doi: 10.1002/eji.70099 (PMC12717635; doi:10.1002/eji.70099)
Supplement: Supplementary file 1 — Supporting File 1: eji70099‐sup‐0001‐SuppMat.pdf. [file EJI-55-e70099-s001.pdf]

Innate						
**Fluorochrome**	**BV605**	**BV711**	**FITC**	**PE**	**PE-DyLight594**	
Target	CRTH2	CD56	CD117	tested molecule	CD3	CD19
Clone	BM16	HCD56	104D2		UCHT1	LT19
**Fluorochrome**	**PerCP-Cy5.5**	**PECy7**	**APC**	**AF700**	**APC-Cy7**	
Target	CD14	CD11c	CD123	HLA-DR	CD16	
Clone	MEM-15	BU15	6H6	L243	3G8	
**Adaptative**						
**Fluorochrome**	**BV605**	**FITC**	**PE**	**PE-Dazzle594**	**PerCP-Cy5.5**	
Target	CXCR5	CD27	tested molecule	CD127	CD4	IgD
Clone	J252D4	LT27		A019D5	MEM-241	IA6-2
**Fluorochrome**	**PECy7**		**APC**	**AF700**	**APC-Cy7**	
Target	TCRgd	CD19	CD25	CD3	CD8	
Clone	B1	LT19	MEM-181	UCHT1	MEM-31	

Supplementary Table 1. Backbone antibodies for subpopulation staining.

CDs	MOLECULE	plasmid	company	GenBank
**CD198**	**CCR8**	pcDNA3.1	GenScript	NM_005201.3
**CD199**	**CCR9**	pUNO1	invivogen	NM_031200.2
**CD372**	**CCR10**	pcDNA3.1	GenScript	NM_016602.3
**CD373**	**CX3CR1**	pcDNA3.1	GenScript	NM_001171174.1
**CD374**	**XCR1**	pcDNA3.1	GenScript	NM_005283.3
**CD375**	**GPR15**	pcDNA3.1	GenScript	NM_005290.4
**CDw376**	**GPR26**	pcDNA3.1	GenScript	NM_153442.4
**CD377**	**SSTR3**	pcDNA3.1	GenScript	NM_001051.5
**CD378**	**C3AR1**	pcDNA3.1	GenScript	NM_004054.4
**CDw379**	**FPR2**	pcDNA3.1	GenScript	XM_006723120.3
**CD380**	**LTB4R**	pcINeo	GenScript	NM_001143919.3
**CDw381**	**GPR183**	pUNO1	invivogen	NM_004951.5
**CDw382**	**F2RL1**	pcDNA3.1	GenScript	NM_005242.5

Supplementary Table 2. Plasmids used for cell transfection.

CDs	MOLECULE	CLONES	Company	VALIDATED OR NOT
**CD198**	**CCR8**	L263G8	Biolegend	VALIDATED
		L263G10	Biolegend	VALIDATED
**CD199**	**CCR9**	L053E8	Biolegend	VALIDATED
		CCR9.1 349	Pablo Engel	VALIDATED
**CD372**	**CCR10**	1B5	BD	VALIDATED
		314305	R&D Systems	VALIDATED
**CD373**	**CX3CR1**	2A9-1	BD	VALIDATED
		K0124E1	Biolegend	VALIDATED
**CD374**	**XCR1**	S15046E	Biolegend	VALIDATED
		S15046A	Biolegend	VALIDATED
		S15046K	Biolegend	VALIDATED
		1097A	R&D Systems	VALIDATED
**CD375**	**GPR15**	SA302A10	Biolegend	VALIDATED
		367902	R&D Systems	VALIDATED
**CDw376**	**GPR26**	891318	R&D Systems	VALIDATED
**CD377**	**SSTR3**	576017	R&D Systems	VALIDATED
		7H8E5	Thermo	VALIDATED
**CD378**	**C3AR1**	hC3aRZ8	BD	VALIDATED
		534625	Thermo	VALIDATED
**CDw379**	**FPR2**	304405	R&D Systems	VALIDATED
**CD380**	**LTB4R**	203_14F11	BD	VALIDATED
		203_14F11	R&D Systems	VALIDATED
		202/7B1	Serotec	VALIDATED
**CDw381**	**GPR183**	SA313E4	Biolegend	VALIDATED
**CDw382**	**F2RL1**	344222	R&D Systems	VALIDATED

Supplementary Table 3. Validated antibody clones.

Supplementary Figure 1

A Target information

Specifity: CCR8 **(CD198)**

clone information

**Clone**	Isotype	Producer	Reactivity
L263G8	mIgG2a, k	BioLegend	human
L263G10	mIgG2a, k	BioLegend	human

B

**Type**	**Cell line**	L263G8	L263G10
Hodgkin Lymphoma	HDLM-2	+	+
Monocytic leukemia	THP-1	-	-
T cell leukemia	Jurkat	-	-
Burkitt lymphoma	Raji	-	-

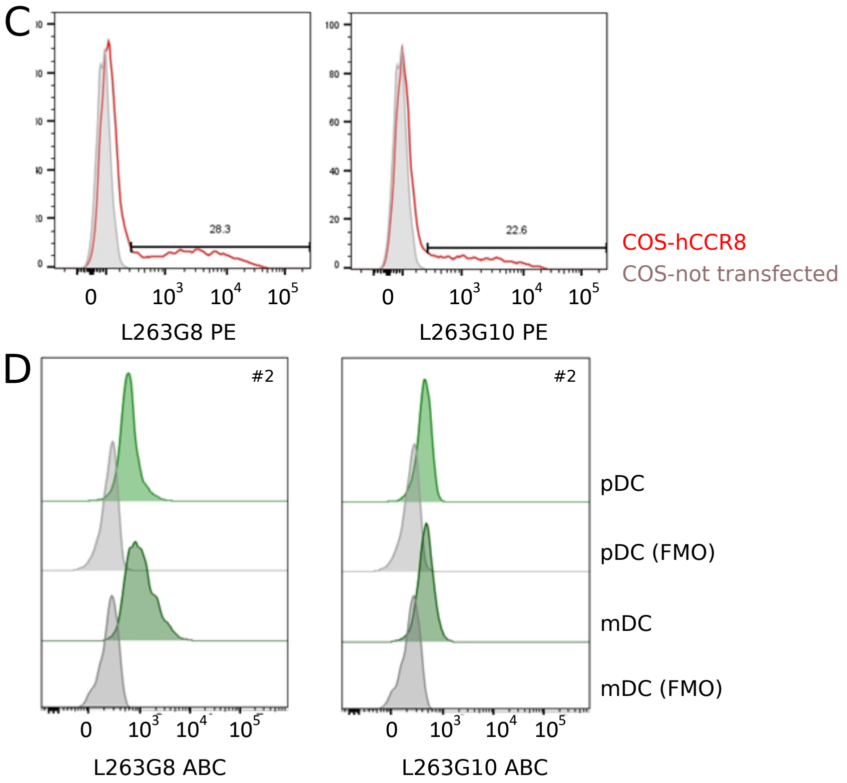

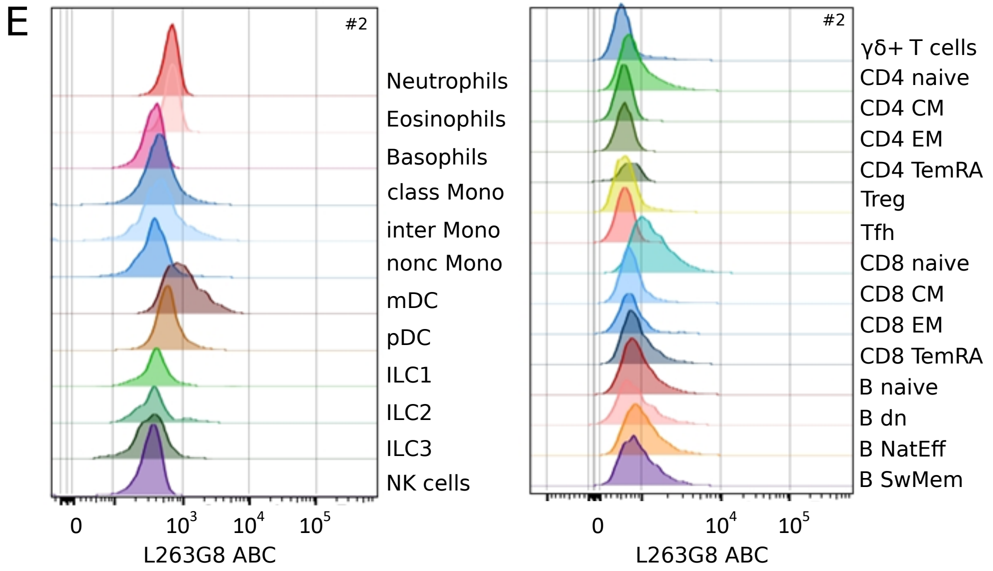

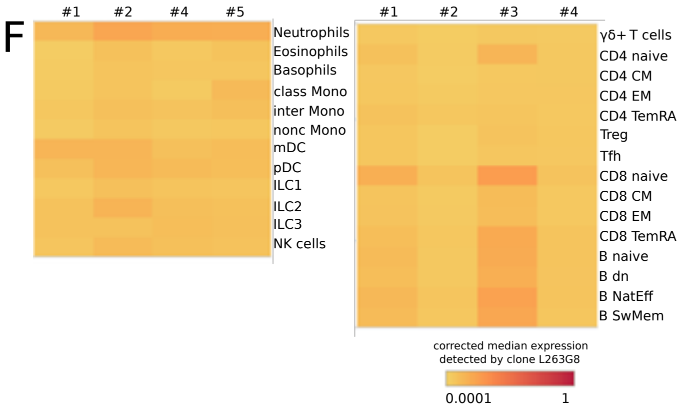

Supplementary Figure 1 Validation data for CD198 mAbs. (A) List of clones, their isotypes, producers and reactivities. (B) Reactivity of validated clones with cell lines (2nd row) representing different cell types (1st row). "+" indicates reactivity of particular clone with the respective cells line; "-" indicates no reactivity. (C) Reactivity of individual clones with COS cells transduced with the *CCR8* cDNA, red lines indicate transduced cells, grey lines indicate non-transduced cells. The x-axis shows fluorescence intensity of PE. (D) Reactivity of individual clones with selected peripheral blood subpopulations. Green lines represent positive cell subset; gray lines represent FMO controls. The x-axis shows ABC. (E) Reactivity of selected clone across 12 innate (left) and 15 adaptive (right) peripheral blood subpopulations. The x-axis shows the ABC of L263G8 clone. (F) Arcsinh-transformed, background-corrected median expression detected by L263G8 clone across 12 innate (left) and 15 adaptive (right) peripheral blood subpopulations (rows) in four healthy donors (columns). ABC - antibody bound per cell; Bdn – double negative B cells; Bnaive – naïve B cells; Bnateff – natural effector B cells; BswMem – switched memory B cells; CM - central memory; classMono – classical monocytes; EM - effector memory; FMO– fluorescence minus one; h – human; ILC1-3 – Innate lymphoid cells 1-3; interMono – intermediate monocytes; m – mouse; mAb – monoclonal antibodies; mDC – myeloid dendritic cells; NK – Natural Killer; noncMono – non-classical monocytes; pDC – plasmacytoid dendritic cells; PE – Phycoerythrine; TemRa – terminal effector memory; Tfh – folicular helper T cells; Treg – regulatory T cells

Supplementary Figure 2

A Target information

Specifity: CCR9 **(CD199)**

clone information

**Clone**	Isotype	Producer	Reactivity
L053E8	mIgG2a, k	BioLegend	human
CCR9.1.349	mIgG2a	Academic (Engel)	human

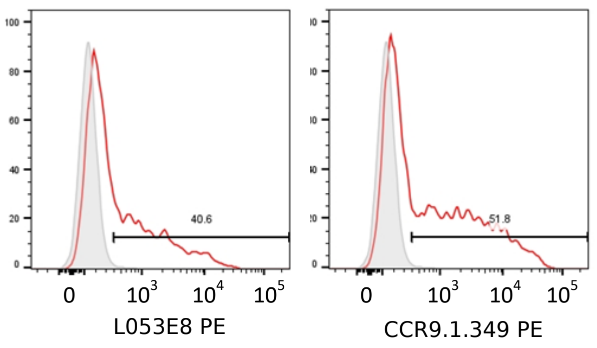

B

**Type**	**Cell line**	L053E8	CCR9.1.349
T cell leukemia	MOLT-4	+	+
Monocytic leukemia	THP-1	-	-
T cell leukemia	Jurkat	-	-
Burkitt lymphoma	Raji	-	-

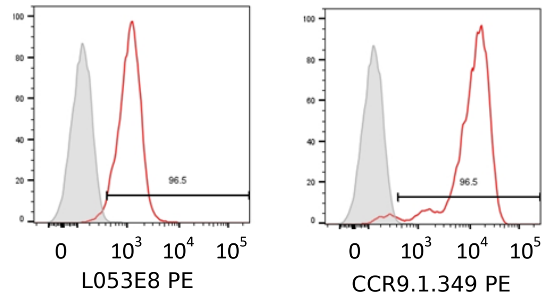

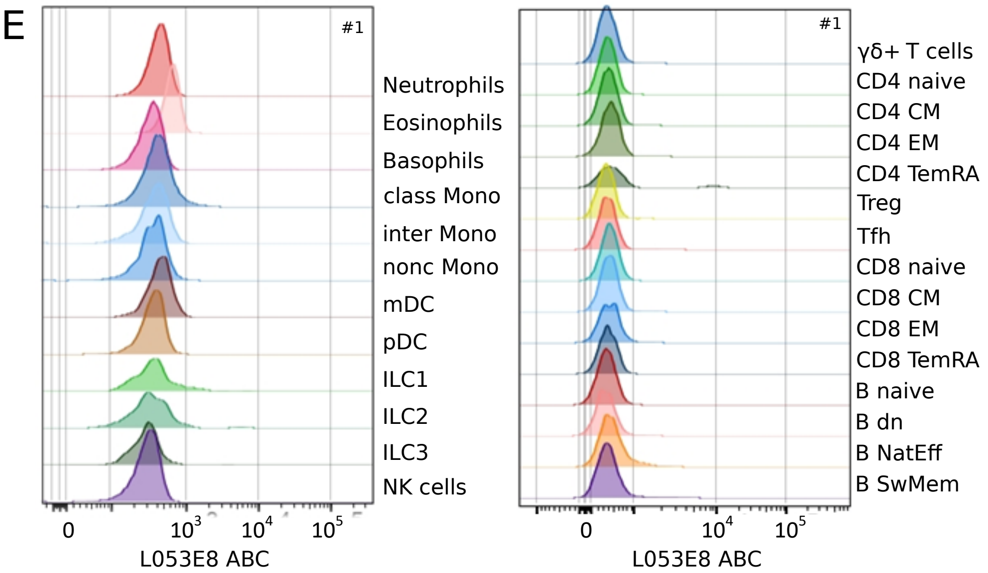

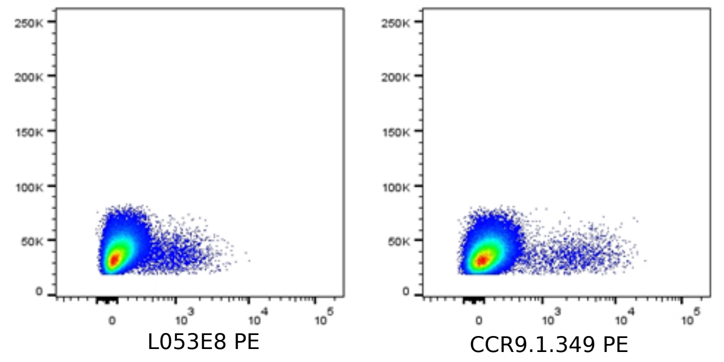

Supplementary Figure 2 Validation data for CD199 mAbs. (A) List of clones, their isotypes, producers and reactivities. (B) Reactivity of validated clones with cell lines (2nd row) representing different cell types (1st row). "+" indicates reactivity of particular clone with the respective cells line; "-" indicates no reactivity. (C) Reactivity of individual clones with COS cells transduced with the *CCR9* cDNA, red lines indicate transduced cells, grey lines indicate non-transduced cells. The x-axis shows fluorescence intensity of PE. (D) Reactivity of individual clones with MOLT4 cell line. Red lines represent stained samples; gray lines represent unstained controls. The x-axis shows the PE. (E) Reactivity of selected clone across 12 innate (left) and 15 adaptive (right) peripheral blood subpopulations. The x-axis shows the ABC of L053E8. (F) Reactivity of individual clones with CD4+ T cell in peripheral blood. ABC - antibody bound per cell; Bdn – double negative B cells; Bnaive – naive B cells; Bnateff – natural effector B cells; BswMem – switched memory B cells; CM - central memory; classMono – classical monocytes; EM - effector memory; FMO– fluorescence minus one; h – human; ILC1-3 – Innate lymphoid cells 1-3; interMono – intermediate monocytes; m – mouse; mAb – monoclonal antibodies; mDC – myeloid dendritic cells; NK – Natural Killer; noncMono – non-classical monocytes; pDC – plasmacytoid dendritic cells; PE – Phycoerythrine; TemRa – terminal effector memory; Tfh – folicular helper T cells; Treg – regulatory T cells

Supplementary Figure 3

A Target information

Specifity: CCR10 **(CD372)**

clone information

**Clone**	Isotype	Producer	Reactivity
1B5	mIgG2a, k	BD Biosciences	human
314305	Rat IgG2a	R&D	human

B

**Type**	**Cell line**	1B5	314305
Multiple myeloma	U266	+	+
Monocytic leukemia	THP-1	+	+
T cell leukemia	Jurkat	-	-
Burkitt lymphoma	Raji	-	-

C

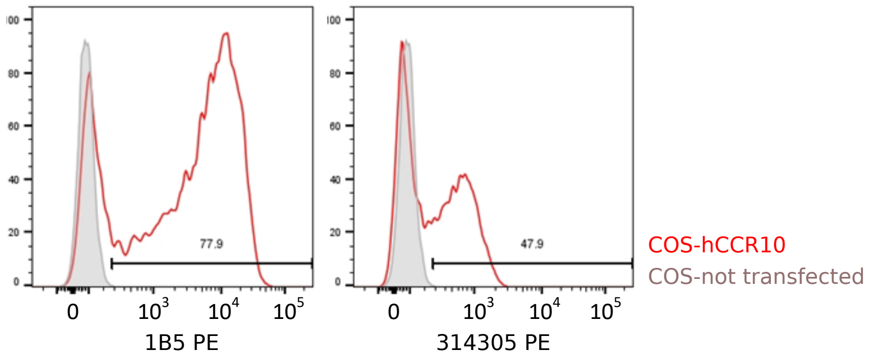

D

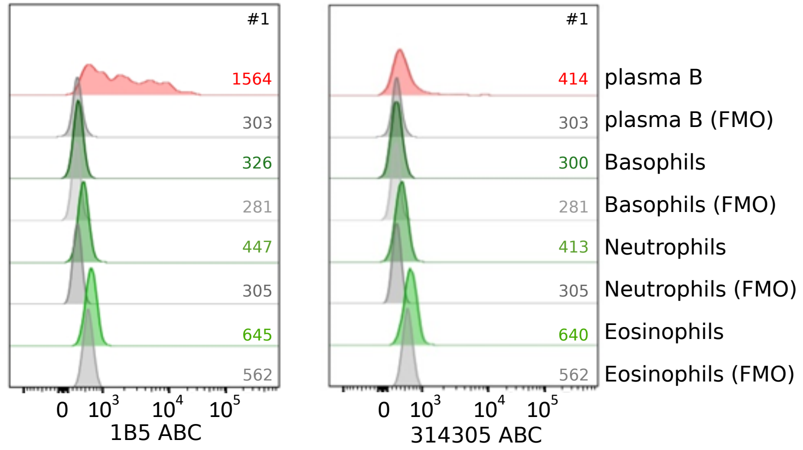

E

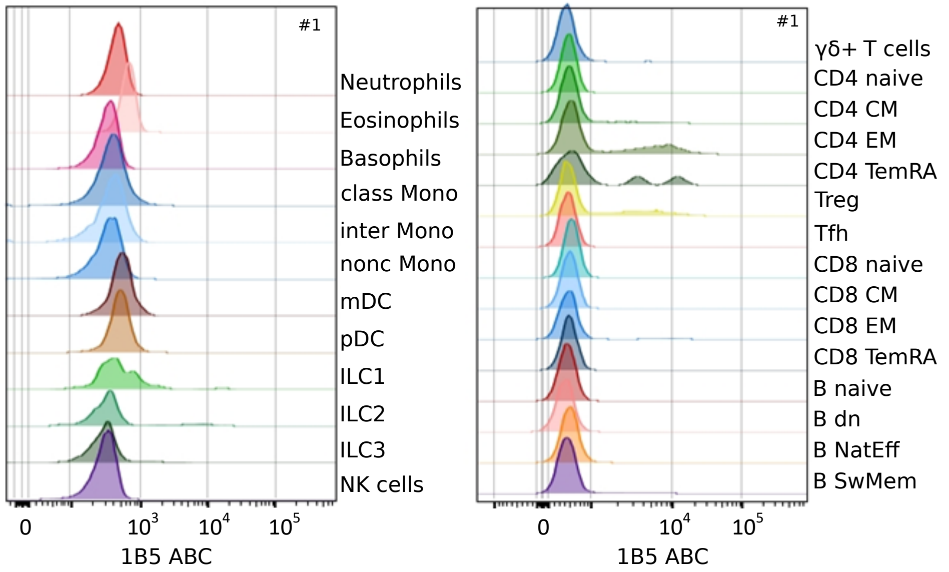

F

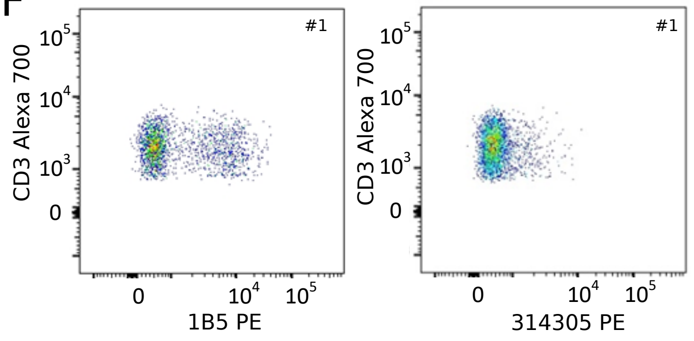

Supplementary Figure 3 Validation data for CD372 mAbs. (A) List of clones, their isotypes, producers and reactivities. (B) Reactivity of validated clones with cell lines (2nd row) representing different cell types (1st row). "+" indicates reactivity of particular clone with the respective cells line; "-" indicates no reactivity. (C) Reactivity of individual clones with COS cells transduced with the *CCR10* cDNA, red lines indicate transduced cells, grey lines indicate non-transduced cells. The x-axis shows fluorescence intensity of PE. (D) Reactivity of individual clones with selected peripheral blood subpopulations. Red lines represent positive cell populations, green lines represent negative cell populations; gray lines represent FMO controls. Numbers represents medians of ABC. The x-axis shows ABC. (E) Reactivity of 1B5 clone across 12 innate (left) and 15 adaptive (right) peripheral blood subpopulations. The x-axis shows the ABC of 1B5 clone. (F) Reactivity of individual clones with CD4 EM T cell in peripheral blood. ABC - antibody bound per cell; Bdn – double negative B cells; Bnaive – naïve B cells; Bnateff – natural effector B cells; BswMem – switched memory B cells; classMono – classical monocytes; CM - central memory; EM - effector memory; FMO– fluorescence minus one; h – human; ILC1-3 – Innate lymphoid cells 1-3; interMono – intermediate monocytes; m – mouse; mDC – myeloid dendritic cells; mAb – monoclonal antibodies; NK – Natural Killer; noncMono – non-classical monocytes; pDC – plasmacytoid dendritic cells; PE – Phycoerythrine; TemRa – terminal effector memory; Tfh – folicular helper T cells; Treg – regulatory T cells

Supplementary Figure 4

A Target information

Specifity: XCR1 **(CD374)**

clone information

**Clone**	Isotype	Producer	Reactivity
S15046E	Rat IgG2a, k	Biolegend	human
S15046A	Rat IgG2a, k	Biolegend	human
S15046K	Rat IgG2a, k	Biolegend	human
1097A	Rb IgG	R&D	human

B

**Type**	**Cell line**	S15046E	S15046A	S15046K	1097A
Multiple myeloma	U266	+	+	+	+
Monocytic leukemia	THP-1	-	-	-	-
T cell leukemia	Jurkat	-	-	-	-
Burkitt lymphoma	Raji	-	-	-	-

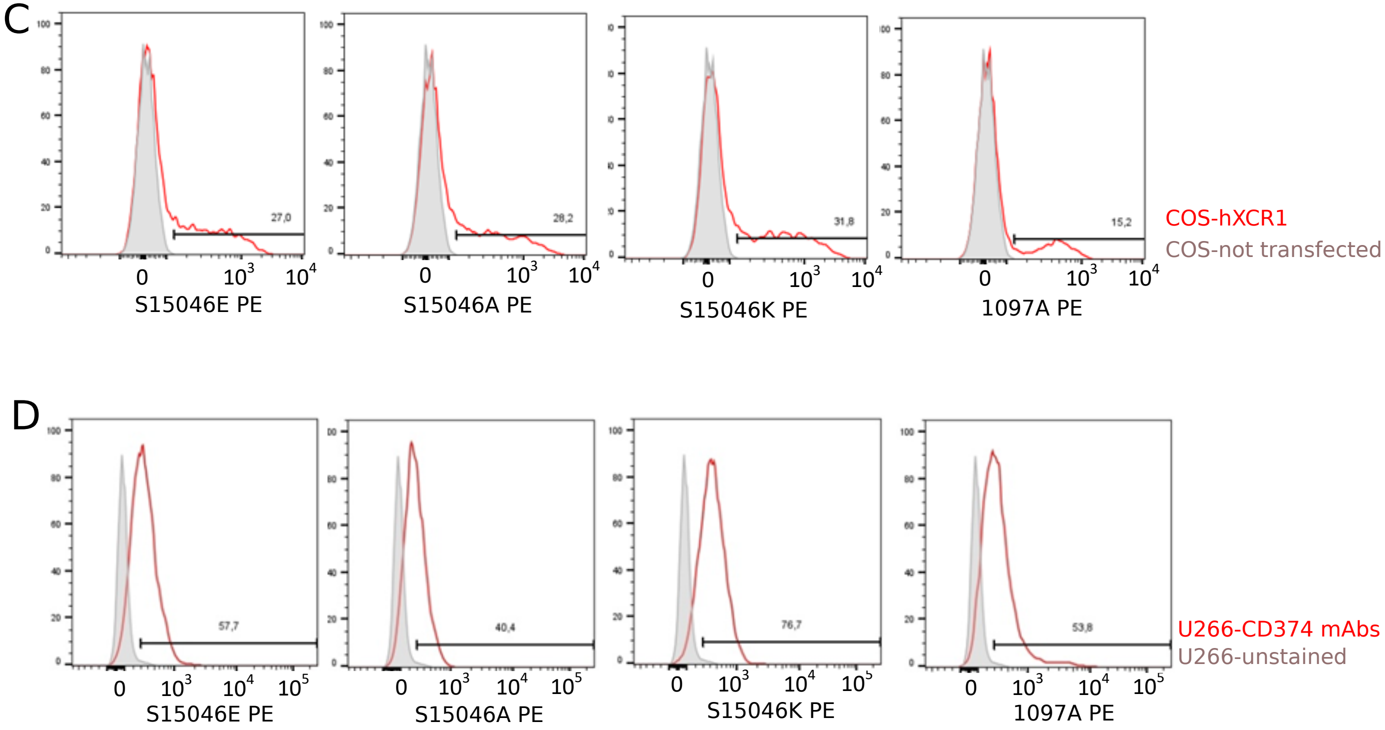

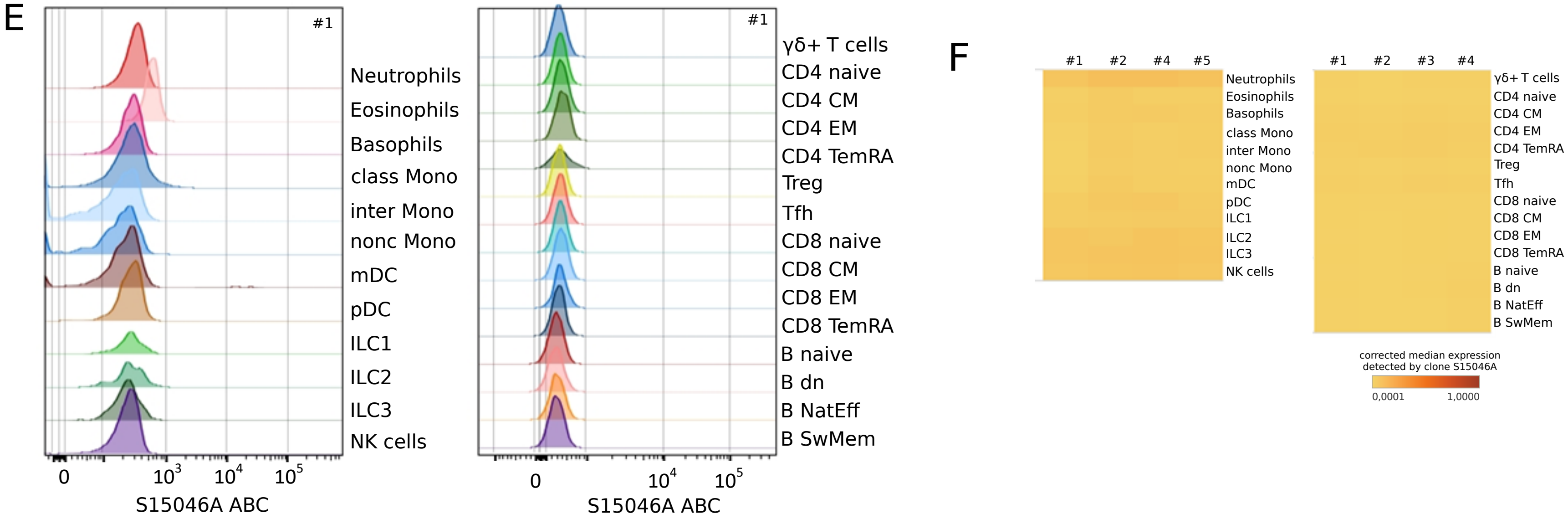

Supplementary Figure 4 Validation data for CD374 mAbs. (A) List of clones, their isotypes, producers and reactivities. (B) Reactivity of validated clones with cell lines (2nd row) representing different cell types (1st row). "+" indicates reactivity of particular clone with the respective cells line; "-" indicates no reactivity. (C) Reactivity of individual clones with COS cells transduced with the *XCR1* cDNA, red lines indicate transduced cells, grey lines indicates non-transduced cells. The x-axis shows fluorescence intensity of PE. (D) Reactivity of individual clones with U266 cell line. Red lines represent stained samples; gray lines represent unstained controls. The x-axis shows PE intensity. (E) Reactivity of S15046A clone across 12 innate (left) and 15 adaptive (right) peripheral blood subpopulations. The x-axis shows the ABC of S15046A. (F) Arcsinh-transformed, background-corrected median expression detected by S15046A clone across 12 innate (left) and 15 adaptive (right) peripheral blood subpopulations (rows) in four healthy donors (columns). ABC - antibody bound per cell; Bdn – double negative B cells; Bnaive – naïve B cells; Bnateff – natural effector B cells; BswMem – switched memory B cells; classMono – classical monocytes; CM - central memory; EM - effector memory; FMO– fluorescence minus one; h – human; ILC1-3 – Innate lymphoid cells 1-3; interMono – intermediate monocytes; mAb – monoclonal antibodies; mDC – myeloid dendritic cells; NK – Natural Killer; noncMono – non-classical monocytes; pDC – plasmacytoid dendritic cells; PE – Phycoerythrine; rb – rabbit; TemRa – terminal effector memory; Tfh – folicular helper T cells; Treg – regulatory T cells

Supplementary Figure 5

A Target information

Specifity: GPR15 **(CD375)**

clone information

**Clone**	Isotype	Producer	Reactivity
SA302A10	mIgG2a, k	BioLegend	human
367902	mIgG2b	R&D	human

B

**Type**	**Cell line**	SA302A10	367902
Chronic B cell leukemia	JVM-2	+	+
Monocytic leukemia	THP-1	-	-
T cell leukemia	Jurkat	-	-
Burkitt lymphoma	Raji	-	-

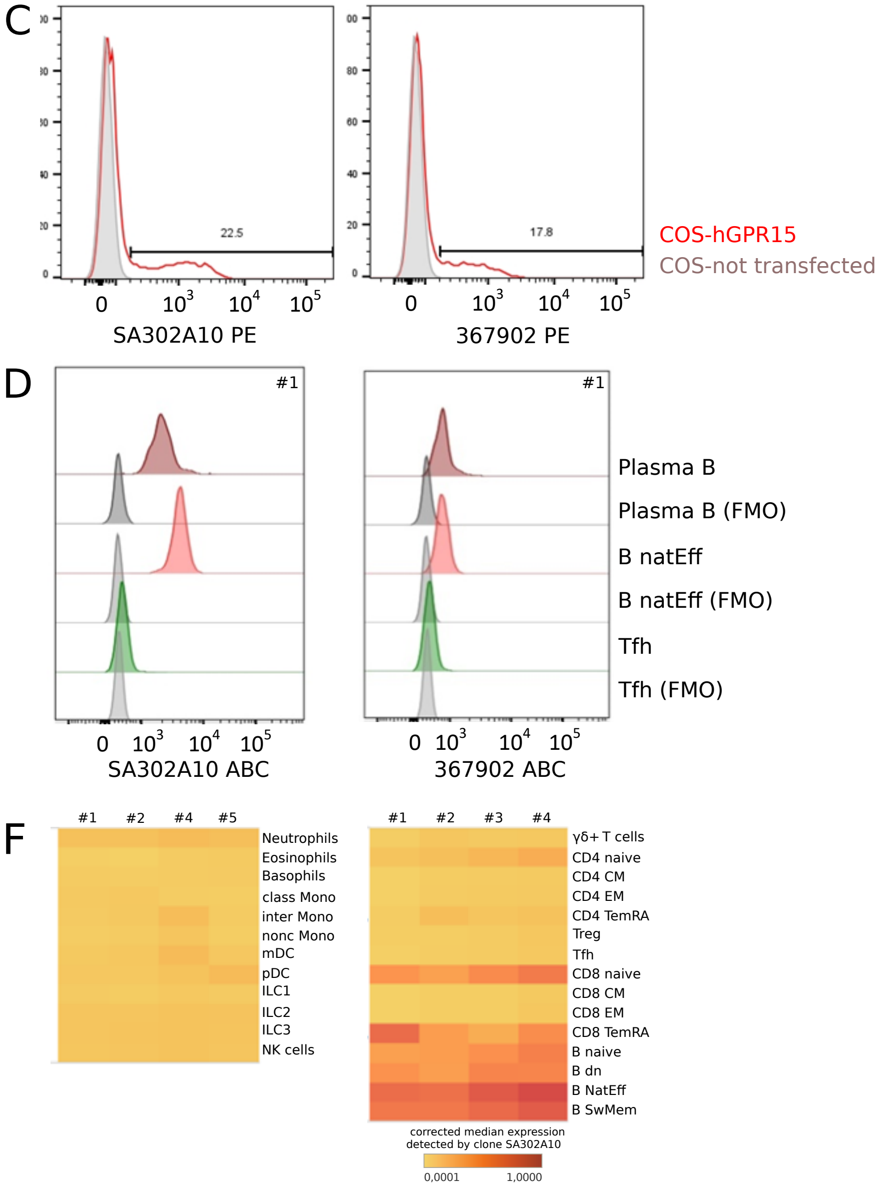

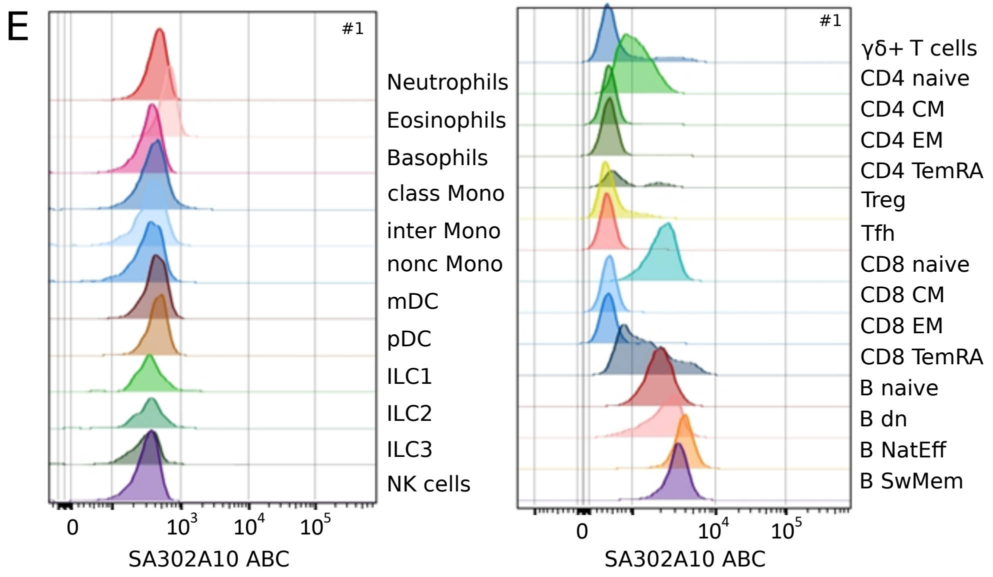

Supplementary Figure 5 Validation data for CD375 mAbs. (A) List of clones, their isotypes, producers and reactivities. (B) Reactivity of validated clones with cell lines (2nd row) representing different cell types (1st row). "+" indicates reactivity of particular clone with the respective cells line; "-" indicates no reactivity. (C) Reactivity of individual clones with COS cells transduced with the *GPR15* cDNA, red lines indicate transduced cells, grey lines indicate non-transduced cells. The x-axis shows fluorescence intensity of PE. (D) Reactivity of individual clones with selected peripheral blood subpopulations. Red lines represent positive subpopulations, green lines represent negative subpopulations, gray lines represent FMO controls. The x-axis shows the ABC intensity. (E) Reactivity of SA302A10 clone across 12 innate (left) and 15 adaptive (right) peripheral blood subpopulations. The x-axis shows the ABC of SA302A10. (F) Arcsinh-transformed, background-corrected median expression detected by SA302A10 clone across 12 innate (left) and 15 adaptive (right) peripheral blood subpopulations (rows) in four healthy donors (columns). ABC - antibody bound per cell; Bdn – double negative B cells; Bnaive – naïve B cells; Bnateff – natural effector B cells; BswMem – switched memory B cells; classMono – classical monocytes; CM - central memory; EM - effector memory; FMO– fluorescence minus one; h – human; ILC1-3 – Innate lymphoid cells 1-3; interMono – intermediate monocytes; m – mouse; mAb – monoclonal antibodies; mDC – myeloid dendritic cells; NK – Natural Killer; noncMono – non-classical monocytes; pDC – plasmacytoid dendritic cells; PE – Phycoerythrine; TemRa – terminal effector memory; Tfh – folicular helper T cells; Treg – regulatory T cells

Supplementary Figure 6

A Target information

Specifity: GPR26 **(CDw376)**

clone information

**Clone**	Isotype	Producer	Reactivity
891318	mIgG2b	R&D	human

B

**Type**	**Cell line**	891318
	no positive cell line	
Monocytic leukemia	THP-1	-
T cell leukemia	Jurkat	-
Burkitt lymphoma	Raji	-

C

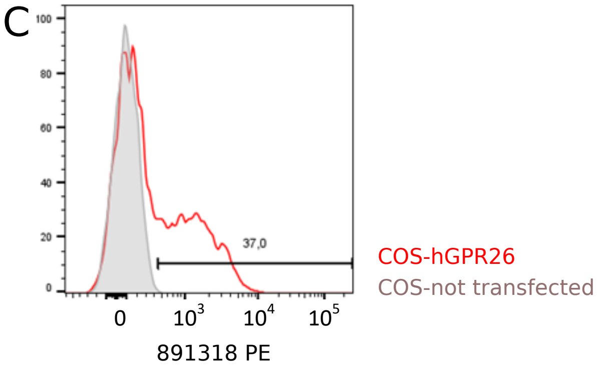

D

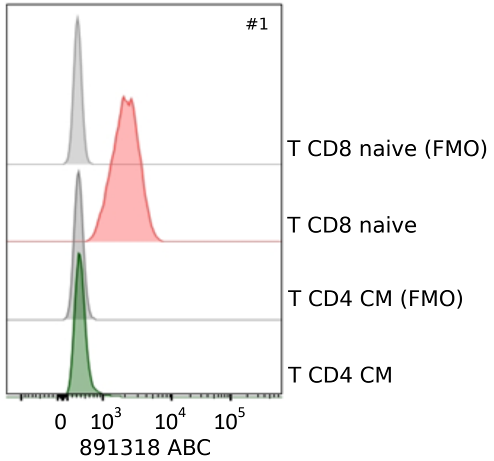

E

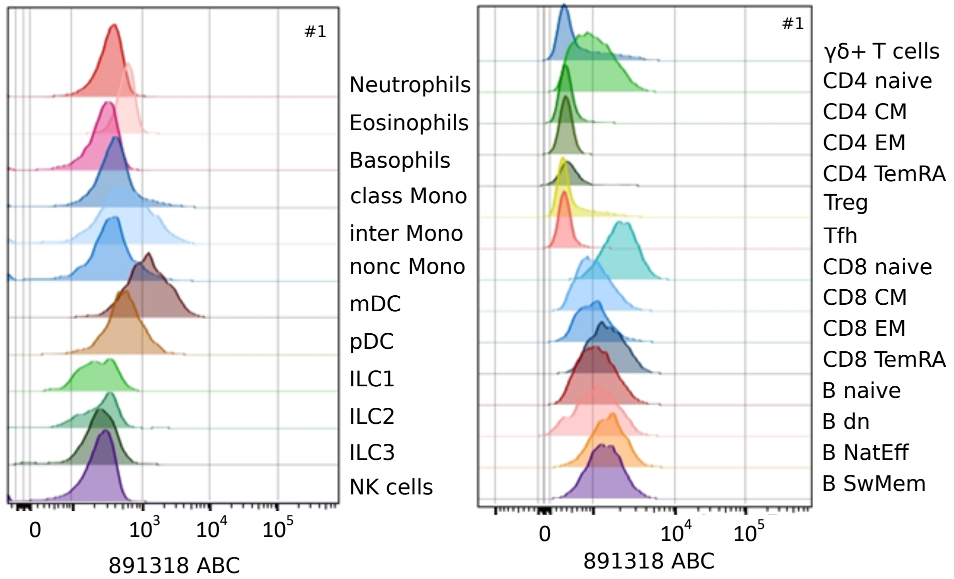

F

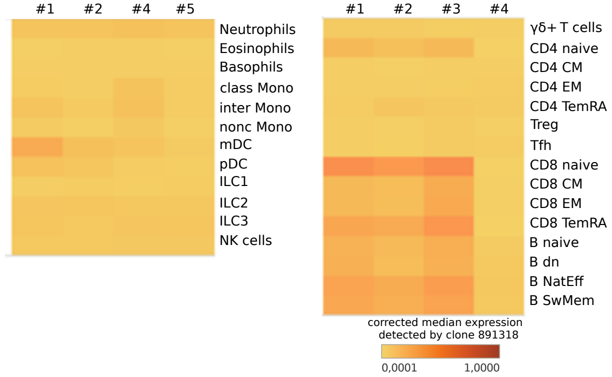

Supplementary Figure 6 Validation data for CDw376 mAb. (A) Clone, its isotype, producer and reactivity. (B) Reactivity of the validated clone with cell lines (2nd row) representing different cell types (1st row). "+" indicates reactivity of the clone with the respective cells line; "-" indicates no reactivity. (C) Reactivity of the clone with COS cells transduced with the *GPR26* cDNA, red line indicates transduced cells, grey line indicates non-transduced cells. The x-axis shows fluorescence intensity of PE. (D) Reactivity of the 891318 clone with selected peripheral blood subpopulations. Red line represents a positive cell subpopulation, green line represents a negative subpopulation, gray lines represent FMO control. The x-axis shows the ABC of 891318. (E) Reactivity of the 891318 clone across 12 innate (left) and 15 adaptive (right) peripheral blood subpopulations. The x-axis shows the ABC of 891318. (F) Arcsinh-transformed, background-corrected median expression detected by the 891318 clone across 12 innate (left) and 15 adaptive (right) peripheral blood subpopulations (rows) in four healthy donors (columns). ABC - antibody bound per cell; Bdn – double negative B cells; Bnaive – naïve B cells; Bnateff – natural effector B cells; BswMem – switched memory B cells; classMono – classical monocytes; CM - central memory; EM - effector memory; FMO– fluorescence minus one; h – human; ILC1-3 – Innate lymphoid cells 1-3; interMono – intermediate monocytes; m – mouse; mAb – monoclonal antibodies; mDC – myeloid dendritic cells; NK – Natural Killer; noncMono – non-classical monocytes; pDC – plasmacytoid dendritic cells; PE – Phycoerythrine; TemRa – terminal effector memory; Tfh – folicular helper T cells; Treg – regulatory T cells

Supplementary Figure 7

A Target information

Specifity: SSTR3 **(CD377)**

clone information

**Clone**	Isotype	Producer	Reactivity
576017	mIgG2a	R&D	human
7H8E5	mIgG1	ThermoFisher	human

B

**Type**	**Cell line**	576017	7H8E5
Multiple myeloma	RPMI8266	+	+
Monocytic leukemia	THP-1	-	-
T cell leukemia	Jurkat	-	-
Burkitt lymphoma	Raji	-	-

C

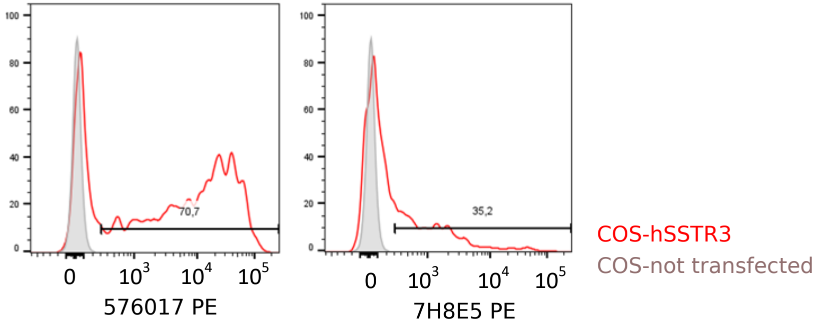

D

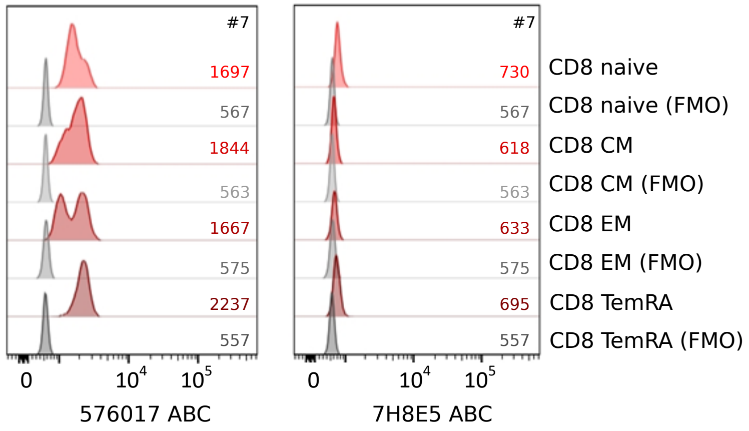

E

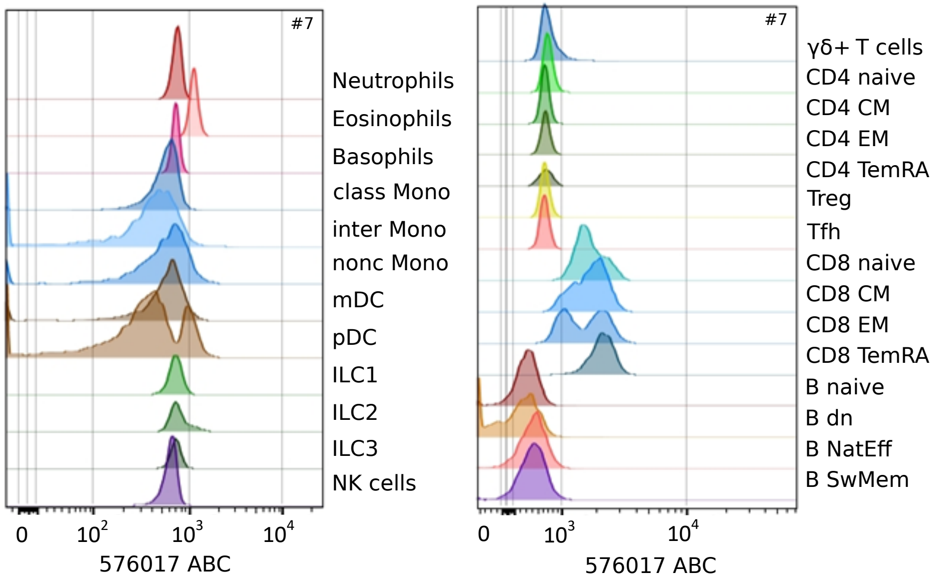

F

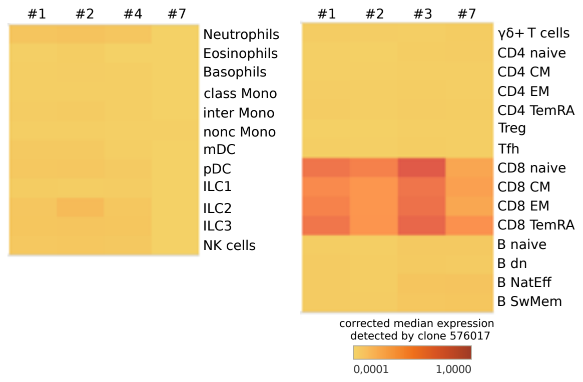

Supplementary Figure 7 Validation data for CD377 mAbs. (A) List of clones, their isotypes, producers and reactivities. (B) Reactivity of validated clones with cell lines (2nd row) representing different cell types (1st row). "+" indicates reactivity of particular clone with the respective cells line; "-" indicates no reactivity. (C) Reactivity of individual clones with COS cells transduced with the *SSTR3* cDNA, red lines indicates transduced cells, grey lines indicates non-transduced cells. The x-axis shows fluorescence intensity of Phycoerythrine (PE). (D) Reactivity of individual clones with selected peripheral blood subpopulations. Red lines represent positive cell subpopulations; gray lines represent FMO controls. Numbers represents medians of ABC. The x-axis shows the ABC. (E) Reactivity of selected clone across 12 innate (left) and 15 adaptive (right) peripheral blood subpopulations. The x-axis shows the ABC of 576017. (F) Arcsinh-transformed, background-corrected median expression detected by 576017 clone across 12 innate (left) and 15 adaptive (right) peripheral blood subpopulations (rows) in four healthy donors (columns). ABC - antibody bound per cell; Bdn – double negative B cells; Bnaive – naïve B cells; Bnateff – natural effector B cells; BswMem – switched memory B cells; classMono – classical monocytes; CM - central memory; EM - effector memory; FMO– fluorescence minus one; h – human; ILC1-3 – Innate lymphoid cells 1-3; interMono – intermediate monocytes; m – mouse; mAb – monoclonal antibodies; mDC – myeloid dendritic cells; NK – Natural Killer; noncMono – non-classical monocytes; pDC – plasmacytoid dendritic cells; PE – Phycoerythrine; TemRa – terminal effector memory; Tfh – folicular helper T cells; Treg – regulatory T cells

Supplementary Figure 8

A Target information

Specifity: C3AR1 **(CD378)**

clone information

**Clone**	Isotype	Producer	Reactivity
hC3aRZ8	mIgG2b	BD Biosciences	human
534625	mIgG2a	ThermoFisher	human

B

**Type**	**Cell line**	hC3aRZ8	534625
Chronic B cell leukemia	JVM-2	+	+
Monocytic leukemia	THP-1	+	+
T cell leukemia	Jurkat	-	-
Burkitt lymphoma	Raji	-	-

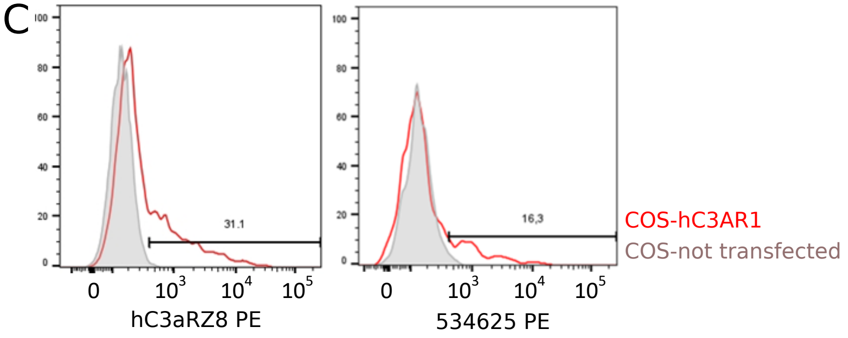

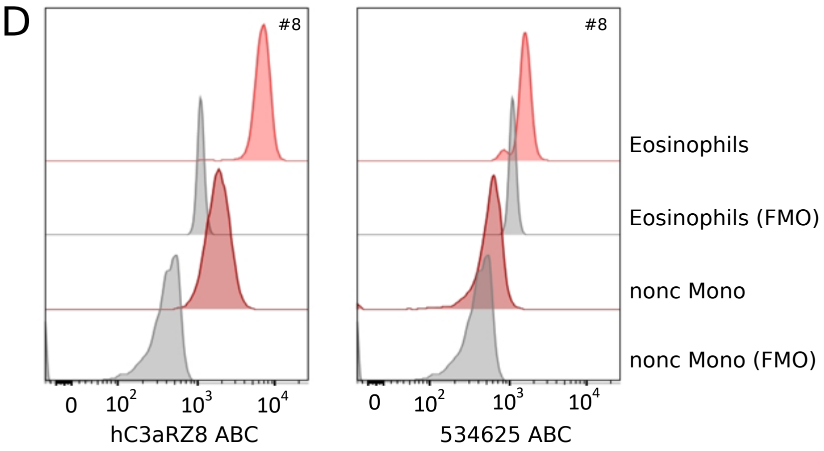

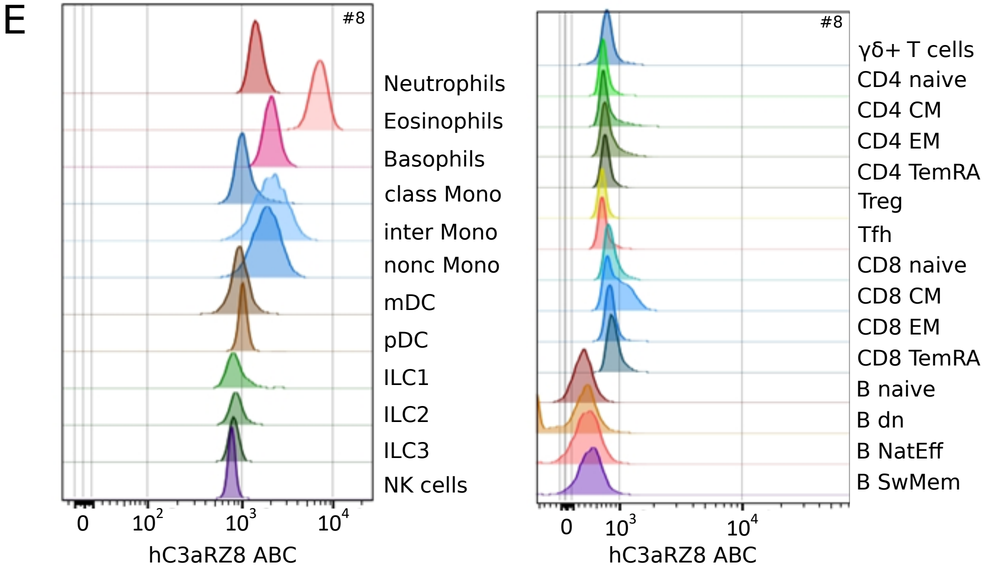

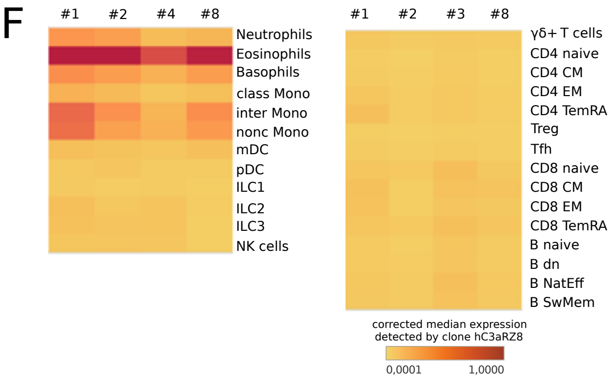

Supplementary Figure 8 Validation data for CD378 mAbs. (A) List of clones, their isotypes, producers and reactivities. (B) Reactivity of validated clones with cell lines (2nd row) representing different cell types (1st row). "+" indicates reactivity of particular clone with the respective cells line; "-" indicates no reactivity. (C) Reactivity of individual clones with COS cells transduced with the *C3AR1* cDNA, red lines indicate transduced cells, grey lines indicate non-transduced cells. The x-axis shows fluorescence intensity of PE. (D) Reactivity of individual clones with selected peripheral blood subpopulations. Red lines represent positive cell subpopulations; gray lines represent FMO controls. The x-axis shows the ABC. (E) Reactivity of hC3aRZ8 clone across 12 innate (left) and 15 adaptive (right) peripheral blood subpopulations. The x-axis shows the ABC.(F) Arcsinh-transformed, background-corrected median expression detected by hC3aRZ8 clone across 12 innate (left) and 15 adaptive (right) peripheral blood subpopulations (rows) in four healthy donors (columns). ABC - antibody bound per cell; Bdn – double negative B cells; Bnaive – naïve B cells; Bnateff – natural effector B cells; BswMem – switched memory B cells; classMono – classical monocytes; CM - central memory; EM - effector memory; FMO– fluorescence minus one; h – human; ILC1-3 – Innate lymphoid cells 1-3; interMono – intermediate monocytes; m – mouse; mAb – monoclonal antibodies; mDC – myeloid dendritic cells; NK – Natural Killer; noncMono – non-classical monocytes; pDC – plasmacytoid dendritic cells; PE – Phycoerythrine; TemRa – terminal effector memory; Tfh – folicular helper T cells; Treg – regulatory T cells

Supplementary Figure 9

A Target information

Specifity: FPR2 **(CDw379)**

clone information

**Clone**	Isotype	Producer	Reactivity
304405	mIgG2b	R&D	human

B

**Type**	**Cell line**	304405
Histiocytic lymphoma	U937	+
Monocytic leukemia	THP-1	-
T cell leukemia	Jurkat	-
Burkitt lymphoma	Raji	-

C

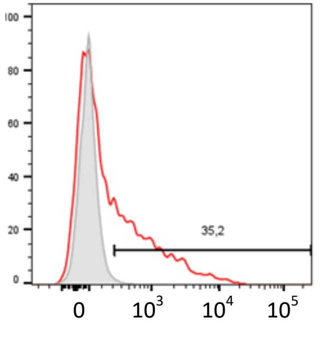

D

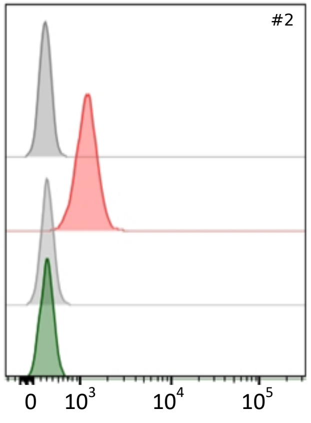

E

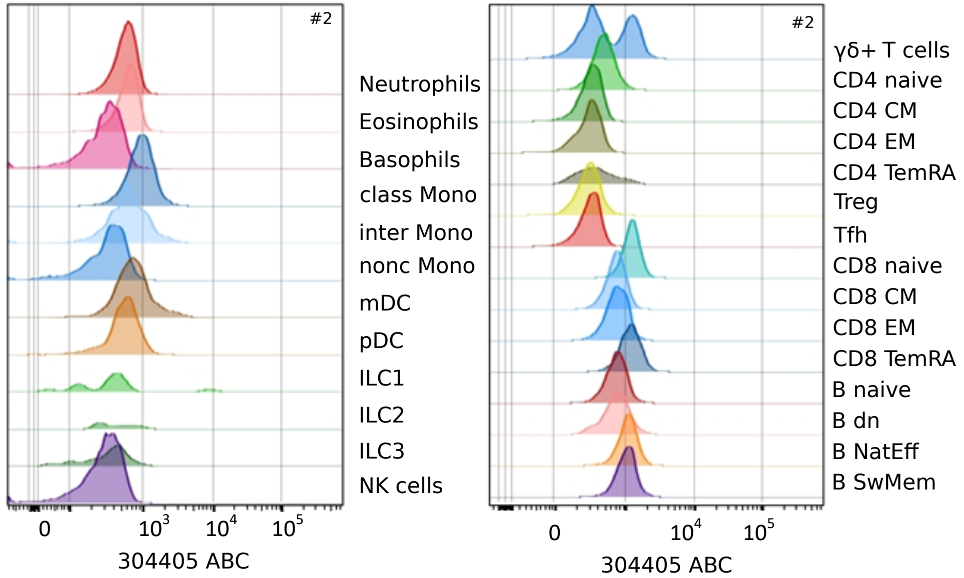

F

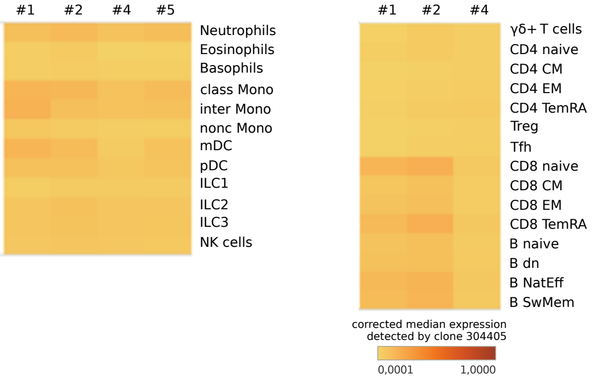

Supplementary Figure 9 Validation data for CDw379 mAb. (A) Clone, its isotype, producer and reactivity. (B) Reactivity of the validated clone with cell lines (2nd row) representing different cell types (1st row). "+" indicates reactivity of the clone with the respective cells line; "-" indicates no reactivity. (C) Reactivity of the clone with COS cells transduced with the *FPR2* cDNA, red line indicates transduced cells, grey line indicates non-transduced cells. The x-axis shows fluorescence intensity of PE. (D) Reactivity of the 304405 clone with selected peripheral blood subpopulations. Red and green lines represent a positive and a negative cell subpopulation, respectively; gray line represents a FMO control. The x-axis shows the ABC. (E) Reactivity of the 304405 clone across 12 innate (left) and 15 adaptive (right) peripheral blood subpopulations. The x-axis shows the ABC.(F) Arcsinh-transformed, background-corrected median expression detected by the 304405 clone across 12 innate (left) and 15 adaptive (right) peripheral blood subpopulations (rows) in four and three, respectively, healthy donors (columns). ABC - antibody bound per cell; Bdn – double negative B cells; Bnaive – naïve B cells; Bnateff – natural effector B cells; BswMem – switched memory B cells; classMono – classical monocytes; CM - central memory; EM - effector memory; FMO– fluorescence minus one; h – human; ILC1-3 – Innate lymphoid cells 1-3; interMono – intermediate monocytes; m – mouse; mAb – monoclonal antibodies; mDC – myeloid dendritic cells; NK – Natural Killer; noncMono – non-classical monocytes; pDC – plasmacytoid dendritic cells; PE – Phycoerythrine; TemRa – terminal effector memory; Tfh – folicular helper T cells; Treg – regulatory T cells

Supplementary Figure 10

A Target information

Specifity: LTB4R **(CD380)**

clone information

**Clone**	Isotype	Producer	Reactivity
203/14F11	mIgG1	BD Biosciences	human
202/7B1	mIgG2a	Serotec	human

B

**Type**	**Cell line**	203/14F11	202/7B1
Histiocytic lymphoma	U937	+	+
Monocytic leukemia	THP-1	-	-
T cell leukemia	Jurkat	-	-
Burkitt lymphoma	Raji	-	-

C

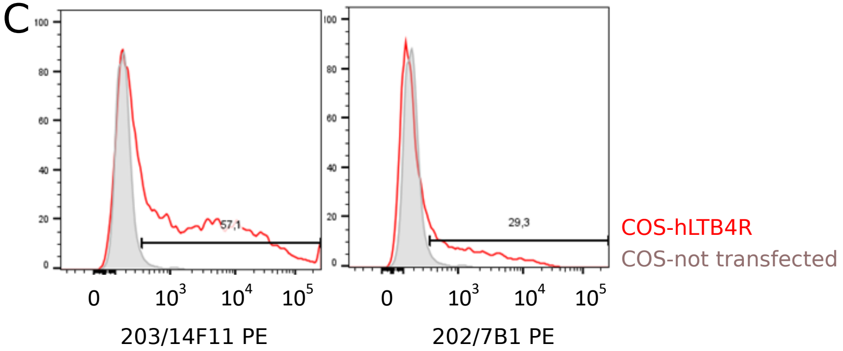

D

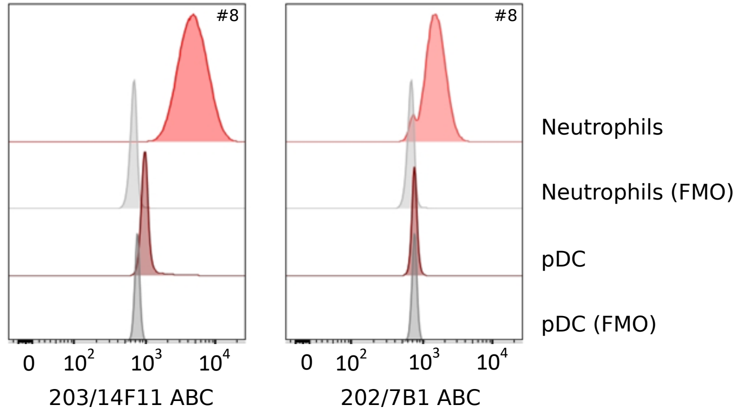

E

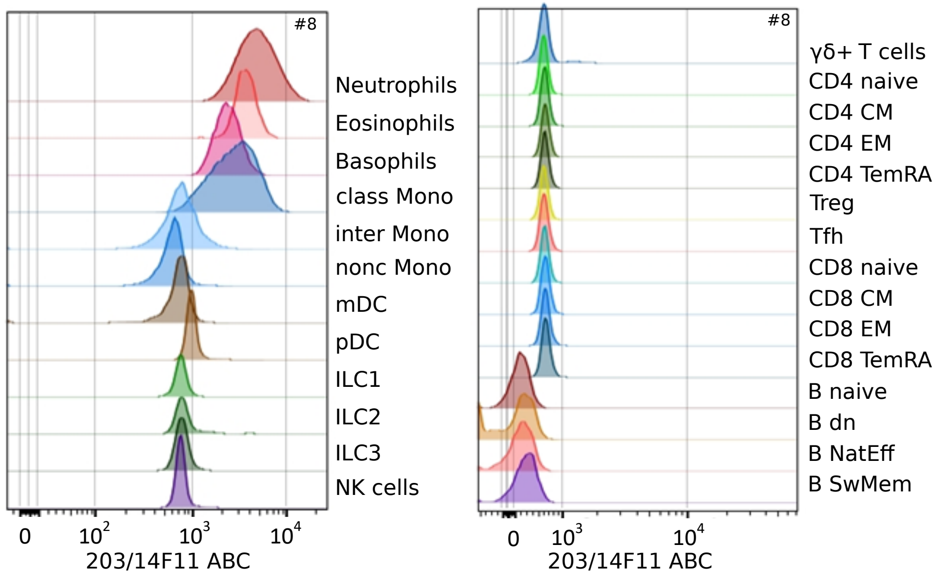

F

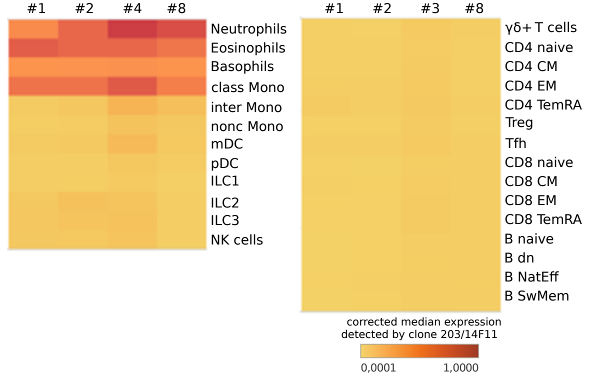

Supplementary Figure 10 Validation data for CD380 mAbs. (A) List of clones, their isotypes, producers and reactivities. (B) Reactivity of validated clones with cell lines (2nd row) representing different cell types (1st row). “+” indicates reactivity of particular clone with the respective cells line; “-“ indicates no reactivity. (C) Reactivity of individual clones with COS cells transduced with the *LTB4R* cDNA, red indicates transduced cells, grey indicates non-transduced cells. The x-axis shows fluorescence intensity of PE. (D) Reactivity of individual clones with selected peripheral blood subpopulations. Red lines represent positive and negative cell subpopulations; gray lines represent FMO controls. The x-axis shows the ABC. (E) Reactivity of 203/14F11 clone across 12 innate (left) and 15 adaptive (right) peripheral blood subpopulations. The x-axis shows the ABC of 203/14F11. (F) Arcsinh-transformed, background-corrected median expression detected by 203/14F11 clone across 12 innate (left) and 15 adaptive (right) peripheral blood subpopulations (rows) in four healthy donors (columns). ABC - antibody bound per cell; Bdn – double negative B cells; Bnaive – naïve B cells; Bnateff – natural effector B cells; BswMem – switched memory B cells; classMono – classical monocytes; CM - central memory; EM - effector memory; FMO– fluorescence minus one; h – human; ILC1-3 – Innate lymphoid cells 1-3; interMono – intermediate monocytes; m – mouse; mAb – monoclonal antibodies; mDC – myeloid dendritic cells; NK – Natural Killer; noncMono – non-classical monocytes; pDC – plasmacytoid dendritic cells; PE – Phycoerythrine; TemRa – terminal effector memory; Tfh – folicular helper T cells; Treg – regulatory T cells

Supplementary Figure 11

A Target information

Specifity: GPR183 **(CDw381)**

clone information

**Clone**	Isotype	Producer	Reactivity
SA313E4	mIgG2a, k	Biolegend	human

B

**Type**	**Cell line**	SA313E4
Hodgkin lymphoma	HLDM-2	+
Monocytic leukemia	THP-1	-
T cell leukemia	Jurkat	-
Burkitt lymphoma	Raji	-

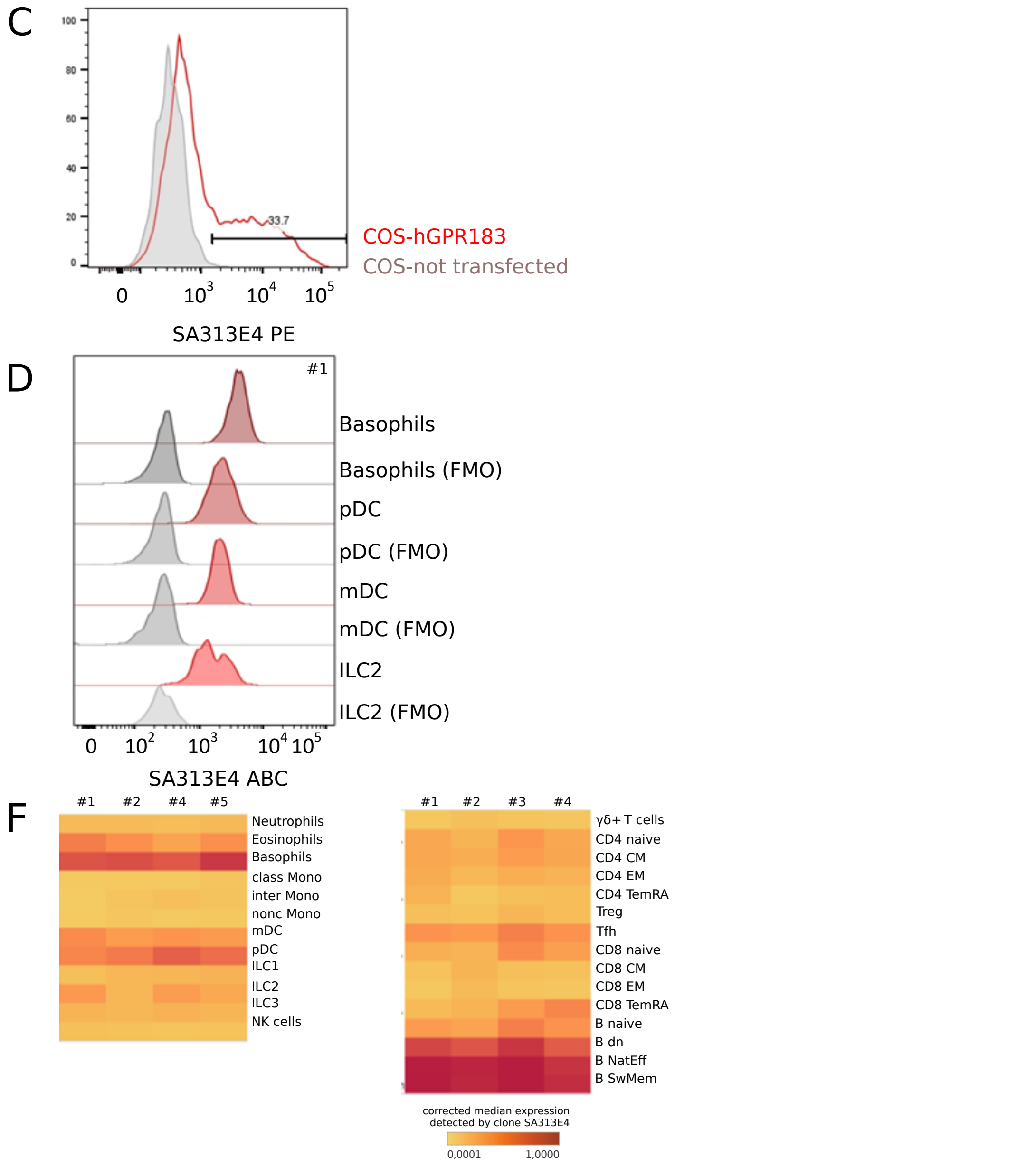

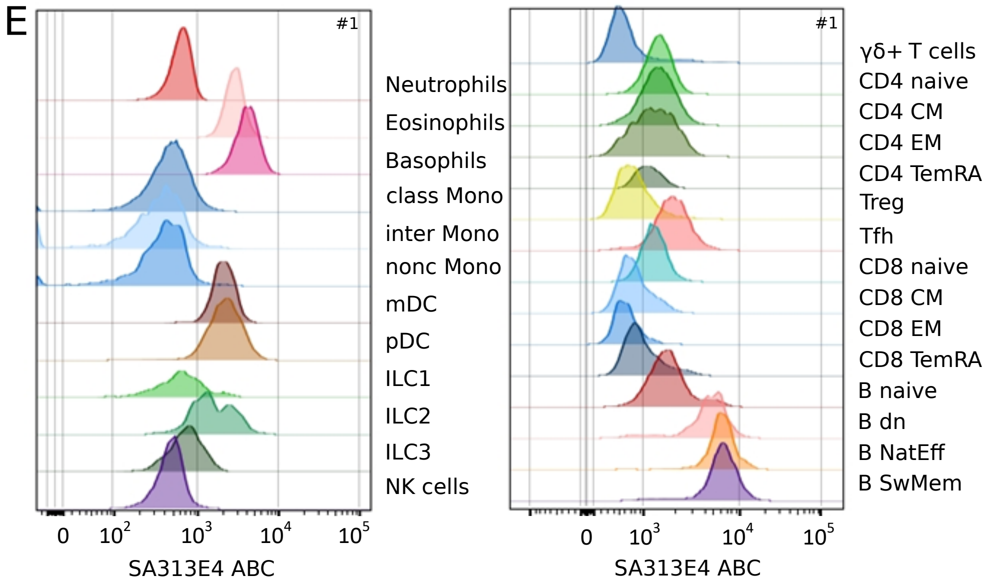

Supplementary Figure 11 Validation data for CDw381 mAb. (A) Clone, its isotype, producer and reactivity. (B) Reactivity of the validated clone with cell lines (2nd row) representing different cell types (1st row). “+” indicates reactivity of the clone with the respective cells line; “-“ indicates no reactivity. (C) Reactivity of the clone with COS cells transduced with the *GPR183* cDNA, red line indicates transduced cells, grey line indicates non-transduced cells. The x-axis shows fluorescence intensity of PE. (D) Reactivity of the SA313E4 clone with selected peripheral blood subpopulations. Red lines represent positive cell subpopulations; gray lines represent FMO controls. The x-axis shows ABC. (E) Reactivity of the SA313E4 clone across 12 innate (left) and 15 adaptive (right) peripheral blood subpopulations. The x-axis shows the ABC.(F) Arcsinh-transformed, background-corrected median expression detected by the SA313E4 clone across 12 innate (left) and 15 adaptive (right) peripheral blood subpopulations (rows) in four healthy donors (columns). ABC - antibody bound per cell; Bdn – double negative B cells; Bnaive – naïve B cells; Bnateff – natural effector B cells; BswMem – switched memory B cells; classMono – classical monocytes; CM - central memory; EM - effector memory; FMO– fluorescence minus one; h – human; ILC1-3 – Innate lymphoid cells 1-3; interMono – intermediate monocytes, mDC – myeloid dendritic cells; m – mouse; mAb – monoclonal antibodies; NK – Natural Killer; noncMono – non-classical monocytes; pDC – plasmacytoid dendritic cells, PE – Phycoerythrine; TemRa – terminal effector memory; Tfh – folicular helper T cells; Treg – regulatory T cells

Supplementary Figure 12

A Target information

Specifity: F2RL1 **(CDw382)**

clone information

**Clone**	Isotype	Producer	Reactivity
344222	mIgG2a	R&D	human

B

**Type**	**Cell line**	344222
T cell leukemia	Jurkat	+
Monocytic leukemia	THP-1	-
Burkitt lymphoma	Raji	-

C

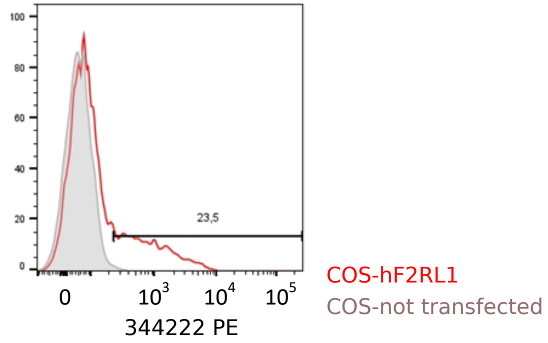

D

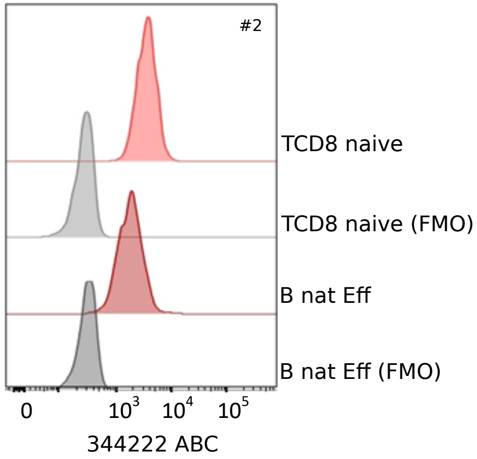

E

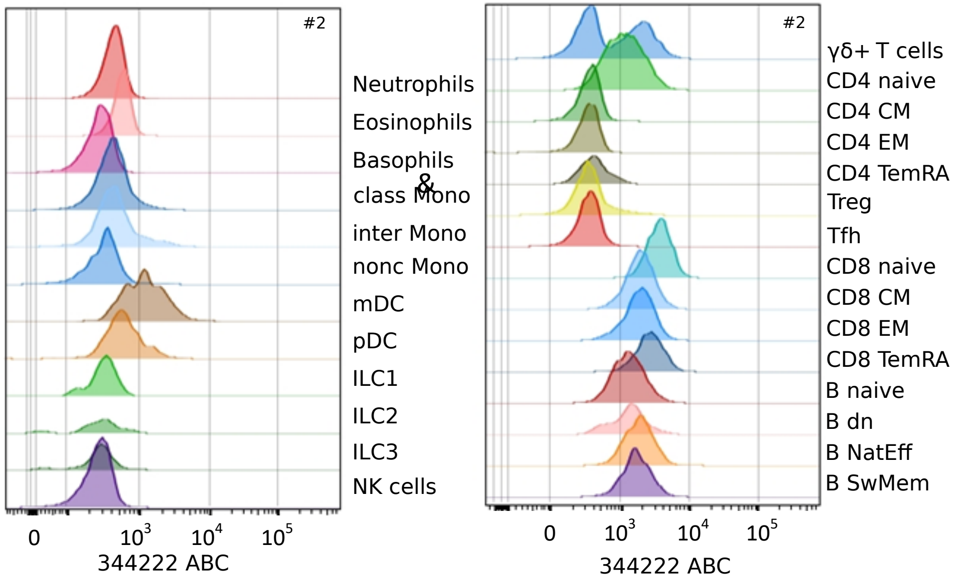

F

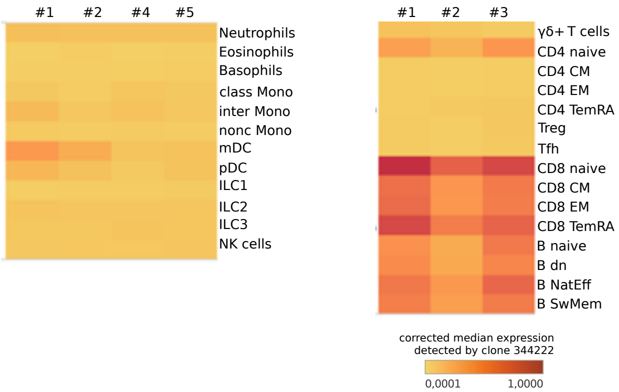

Supplementary Figure 12 Validation data for CDw382 mAb. (A) Clone, its isotype, producer and reactivity. (B) Reactivity of the validated clone with cell lines (2nd row) representing different cell types (1st row). "+" indicates reactivity of the clone with the respective cells line; "-" indicates no reactivity. (C) Reactivity of the clone with COS cells transduced with the *F2RL1* cDNA, red line indicates transduced cells, grey line indicates non-transduced cells. The x-axis shows fluorescence intensity of PE. (D) Reactivity of the 344222 clone with selected peripheral blood subpopulations. Red lines represent positive cell subpopulations; gray lines represent FMO controls. The x-axis shows the ABC. (E) Reactivity of the 344222 clone across 12 innate (left) and 15 adaptive (right) peripheral blood subpopulations. The x-axis shows the ABC. (F) Arcsinh-transformed, background-corrected median expression detected by the 344222 clone across 12 innate (left) and 15 adaptive (right) peripheral blood subpopulations (rows) in four and three, respectively, healthy donors (columns). ABC - antibody bound per cell; Bdn – double negative B cells; Bnaive – naïve B cells; Bnateff – natural effector B cells; BswMem – switched memory B cells; classMono – classical monocytes; CM - central memory; EM - effector memory; FMO– fluorescence minus one; h – human; ILC1-3 – Innate lymphoid cells 1-3; interMono – intermediate monocytes; m – mouse; mAb – monoclonal antibodies; mDC – myeloid dendritic cells; NK – Natural Killer; noncMono – non-classical monocytes; pDC – plasmacytoid dendritic cells; PE – Phycoerythrine; TemRa – terminal effector memory; Tfh – folicular helper T cells; Treg – regulatory T cells

Supplementary Figure 13

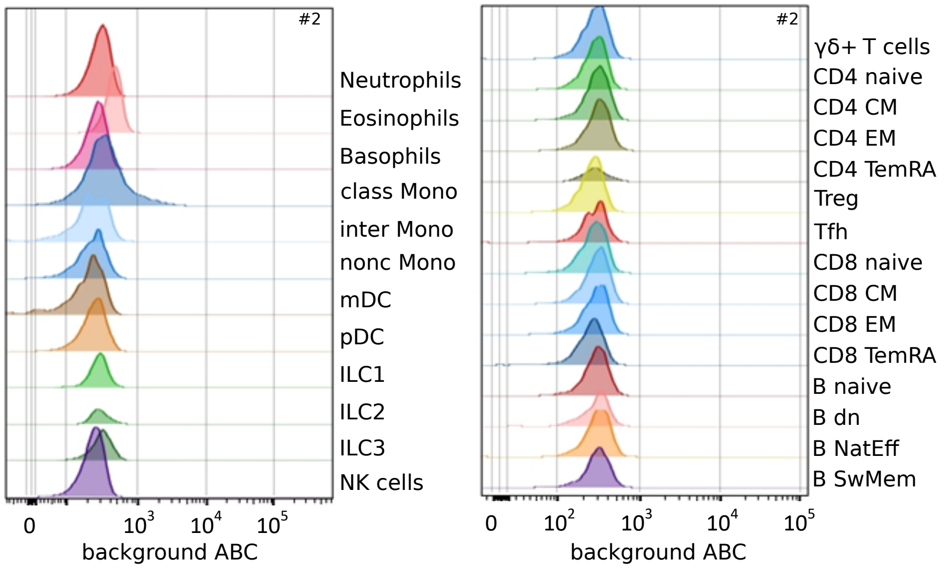

Supplementary Figure 13 Background fluorescence of FMO control across 12 innate (left) and 15 adaptive (right) peripheral blood subpopulations (rows). The x-axis shows the ABC with no mAb in the PE channel used. ABC - antibody bound per cell; Bdn – double negative B cells; Bnaive – naïve B cells; Bnateff – natural effector B cells; BswMem – switched memory B cells; classMono – classical monocytes; CM - central memory; EM - effector memory; FMO– fluorescence minus one; ILC1-3 – Innate lymphoid cells 1-3; interMono – intermediate monocytes; mAb – monoclonal antibodies; mDC – myeloid dendritic cells; NK – Natural Killer; noncMono – non-classical monocytes; pDC – plasmacytoid dendritic cells; PE – Phycoerythrine; TemRa – terminal effector memory; Tfh – folicular helper T cells; Treg – regulatory T cells
